# Bioinspired Adaptive Surfaces for Intelligent Liquid Manipulation: Progressing From Passive and Active to Hybrid Strategies

**DOI:** 10.1002/adma.74360

**Published:** 2026-08-06

**Authors:** Jiaqi Miao, Yiyuan Zhang, Liqiu Wang, Alan C. H. Tsang

**Affiliations:** ^1^ Department of Mechanical Engineering The University of Hong Kong Hong Kong China; ^2^ Department of Mechanical Engineering The Hong Kong Polytechnic University Hong Kong China

**Keywords:** bioinspired surfaces, external fields, hybrid systems, interfacial materials, liquid manipulation, wettability

## Abstract

Natural surfaces demonstrate sophisticated liquid manipulation through passive interfacial energy landscapes, whereas active systems rely on external stimuli for dynamic control. Bringing these strategies offers a promising route to balance energy efficiency with adaptive control. This review first outlines the principles of passive strategies and externally actuated active methods, and then critically assesses emerging hybrid approaches. Hybrid strategies utilize external energy to program the interfacial properties of passive surfaces, after which liquid motion proceeds without continuous energy input, driven by the stored interfacial energy of reconstructed surfaces. This approach, combining passive energy efficiency with active flexibility, could address the trade‐off between low energy consumption and adaptable liquid manipulation. Emerging applications are further discussed across sustainable energy, environmental science, thermal management, smart manufacturing, and healthcare engineering, with guidance on selecting appropriate liquid manipulation strategies for different application scenarios. Finally, perspectives are provided on energy acquisition, material innovation, design methodologies, and system integration, envisioning a technological framework in which passive, active, and hybrid strategies advance in parallel as complementary pillars to accelerate the translation of liquid manipulation surfaces from laboratory discoveries to real‐world applications.

## Graphical abstract

This review examines passive, active, and hybrid liquid manipulation strategies, highlighting hybrid approaches as an emerging route to reconcile energy efficiency with adaptive control. By actively reconstructing passive surfaces to store programmable interfacial energy, hybrid systems enable flexible yet low‐power liquid transport, with perspectives spanning design methodologies, technological translation, and interdisciplinary applications.

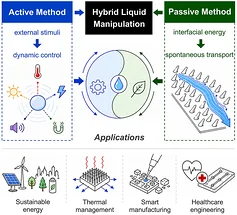

## 1 Introduction

Liquid manipulation is at the forefront of technological innovation, serving as a cornerstone capability that bridges fundamental science and transformative real‐world applications [1, 2, 3, 4, 5, 6]. Its broad interdisciplinary appeal is driving progress across different fields, including atmospheric water harvesting [7, 8, 9, 10], solar‐driven water purification [11, 12], and oil–water separation systems [13, 14]. The impact of liquid manipulation also extends to high‐efficiency chemical processing [15], next‐generation thermal management [16, 17], miniaturized lab‐on‐a‐chip diagnostics [18, 19], wearable devices [20, 21], and biomedical microrobots [22, 23], which ultimately reshape the future technological landscape.

The understanding of interfacial liquid behavior arises from a sequence of fundamental discoveries (Figure 1). Early developments include Leonardo da Vinci's observation of capillarity in the 15th century, Jurin's Law (1718) relating capillary rise to tube radius [24], and Segner's identification of liquid surface tension in 1751 [25]. These advances culminated in the Young–Laplace description of capillary pressure, surface tension, and curvature (1805‐1806) [26] and the subsequent Young–Laplace–Gauss equation unified by Gauss in 1830 [27]. The mid‐19th century then introduces dynamic aspects: the “tears of wine” phenomenon (1855) [28] is later explained by the Marangoni effect (1865) [29], which describes flow driven by surface tension gradients. This is followed by 20th‐century advances in describing interfacial wetting: the Wenzel equation (1936) describes the effect of surface roughness on wettability [30], and Cassie and Baxter (1944) extend this by modeling surfaces with trapped air, that is, the Cassie–Baxter state [31]. These milestones collectively establish the foundational framework for interfacial liquid dynamics.

**FIGURE 1:**
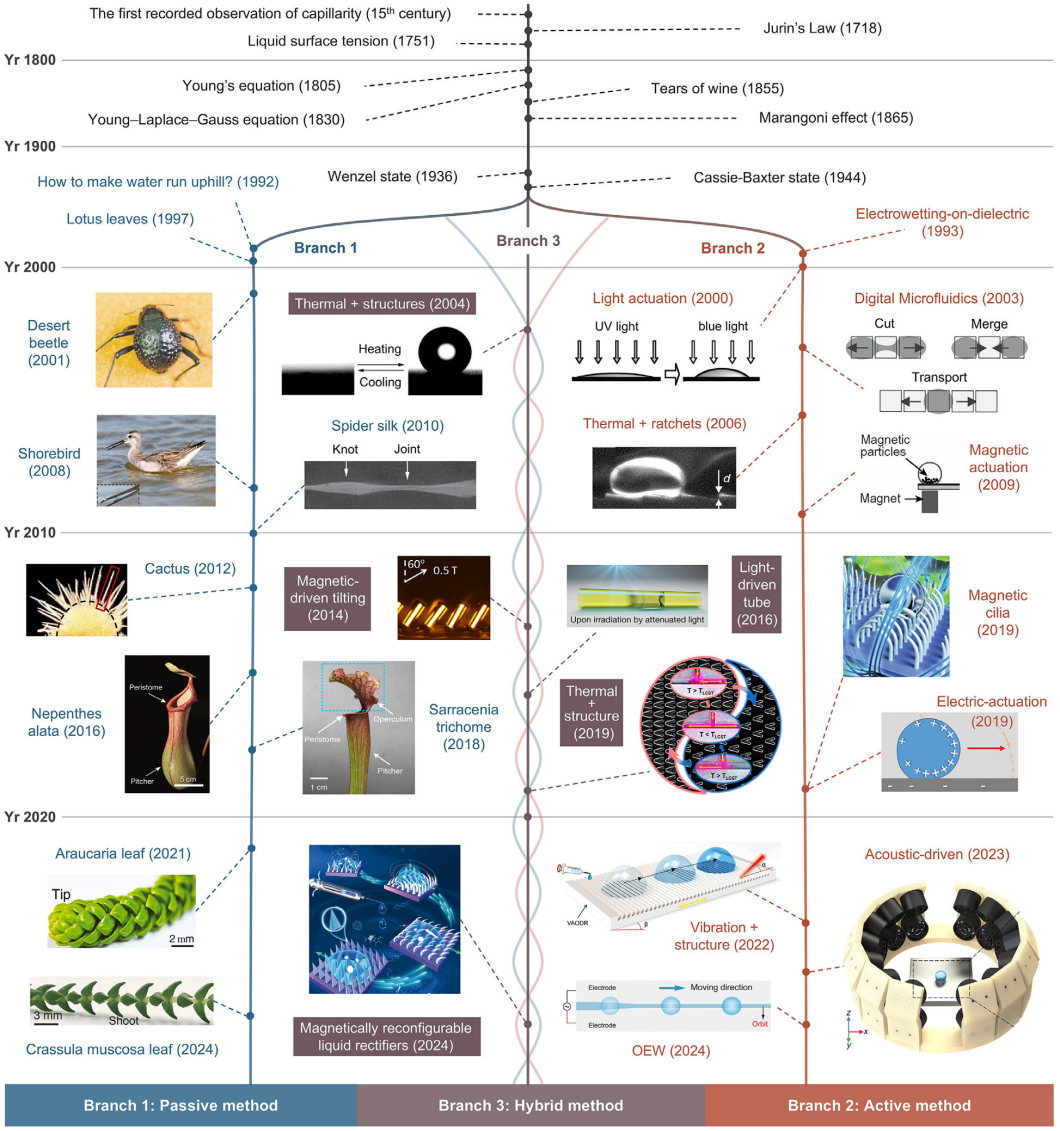
Illustration of the conceptual framework of intelligent liquid manipulation strategies discussed in this review. The field is organized into three interconnected branches: passive surfaces based on bioinspired structures and heterogeneous wettability designs (branch 1), active liquid manipulation driven by electric, magnetic, optical, thermal, or acoustic stimuli (branch 2), and emerging hybrid active–passive systems that integrate programmable field control with interfacial‐energy‐driven transport (branch 3). Reproduced with permission [33, 34, 36, 37, 38, 69]. Copyright 2001/2010/2012/2016/2018, Springer Nature. Reproduced with permission [35, 39, 40, 62, 63]. Copyright 2000/2008/2021/2023/2024, AAAS. Reproduced with permission [60]. Copyright 2009, Royal Society of Chemistry. Reproduced with permission [58, 61, 65, 66, 67, 68, 71]. Copyright 2004/2014/2019/2022/2024, Wiley‐VCH. Reproduced with permission [64]. Copyright 2006, American Physical Society. Reproduced with permission [70]. Copyright 2019, American Chemical Society.

Advanced characterization techniques directly correlate interfacial performance with micro‐/nanoscale topological features, revealing how numerous species have evolved structured surfaces to regulate droplet morphology, wetting states, and dynamic behavior without energy input. As illustrated in Figure 1 (branch 1), notable examples include the self‐cleaning lotus leaf [32], water harvesting methods of desert beetles and cacti [33, 34], drinking strategies of shorebirds [35], water collection by spider silk [36], the rapid liquid transport on *Nepenthes alata* and *Sarracenia* trichomes [37, 38], and reverse liquid behaviors on *Araucaria* and *Crassula muscosa* leaves [39, 40]. These have inspired the design of functional surfaces via techniques such as femtosecond laser processing [41, 42, 43], photolithography [44], etching [45], 3D printing [46, 47, 48, 49], and electrospinning [50]. The engineered surfaces, endowed with specific physical structures [51, 52] or chemical modifications [53, 54] that yield tailored wettability, are ideal for self‐cleaning, anti‐icing, microfluidics, and water harvesting, due to their exceptional energy efficiency and operational autonomy. However, their inherent static design limits post‐fabrication adaptability and multi‐functionality, and thus hinders their operation in changing environments or execution of complex tasks.

In contrast to passive paradigms, active liquid manipulation has emerged as a complementary approach (branch 2 in Figure 1) by employing stimuli‐responsive materials to create dynamic surfaces with on‐demand tunable properties [55]. This active strategy employs various external fields (e.g., electrical [56, 57, 58], thermal [59], magnetic [60, 61], optical [62], and acoustic [63]) to achieve noncontact, flexible, and precise liquid manipulation. These active systems overcome the inherent static limitations of passive surfaces by introducing dynamic control, but their enhanced adaptability often comes at the cost of continuous energy input, increased system complexity, and challenges in scalability in practical contexts.

The inherent divergence between passive and active systems has historically led to two distinct technological pathways. Recognizing their complementary advantages, recent studies have sought to combine these approaches. In such systems, passive architectures are integrated with active actuation to enhance control performance. For example, passive microstructures can guide and direct liquid motion, improving the efficiency, precision, and controllability of externally driven liquid transport [64, 65, 66]. Although these approaches demonstrate useful combinations of passive structures and active inputs, they still rely on continuous external actuation and remain within the active control paradigm. These developments can thus be regarded as preliminary explorations toward hybrid liquid manipulation.

Beyond these preliminary combinations, distinct hybrid strategies have begun to emerge (branch 3 in Figure 1) [67, 68, 69, 70, 71]. In this paradigm, external energy is primarily used to program the static interfacial properties of passive structures, after which liquid motion proceeds autonomously without sustained energy input, driven by interfacial energy stored in the programmed surface. This programming–execution decoupling offers a route to reconcile the long‐standing trade‐off between the energy efficiency of passive systems and the adaptability of active control. Despite these advances, the conceptual boundaries and design principles of hybrid systems remain insufficiently clarified. Compared with the extensive literature on purely passive or active liquid manipulation strategies [1, 2, 3, 4, 72, 73, 74, 75, 76, 77, 78, 79, 80, 81, 82], a systematic and dedicated review of this rapidly developing direction is still lacking.

As summarized in Figure 1, the review is organized around three interconnected branches (passive, active, and hybrid strategies) which evolve in parallel while encompassing distinct principles, control methods, and applications. Rather than implying a strict technological progression, this framework summarizes representative approaches rooted in biomimetic design, external actuation, and passive–active coupling. First, we introduce the fundamental principles of wetting and liquid transport to establish a common framework, then examine passive and active liquid control strategies to clarify their respective advantages and limitations. We next focus on hybrid systems, including defining true hybrid active–passive operation, in which external energy programs interfacial properties while liquid motion is subsequently executed through stored interfacial energy. We then discuss emerging applications in sustainable energy, environmental science, thermal management, smart manufacturing, and healthcare engineering, and provide guidance on selecting appropriate strategies for practical implementation. Finally, we identify future opportunities in energy acquisition, material innovation, design methodologies, and system integration.

## 2 Fundamentals of Interfacial Liquid Control

The intricate manipulation of liquids in natural systems and engineered surfaces is grounded in the physics of interfacial phenomena [83, 84]. A solid grasp of these principles is crucial for designing liquid manipulation surfaces. This section offers an overview of key interfacial wetting mechanisms, beginning with fundamental definitions of wettability and advancing to interfacial Laplace pressure and capillary‐driven transport.

Wettability is central to liquid manipulation, quantitatively described by the contact angle (CA), θ, at the solid–liquid–gas three‐phase contact line (Figure 2A). For an idealized, smooth, homogeneous, and rigid surface, CA is determined by a balance of interfacial energies and expressed by Young's equation [26],

(1) $$\gamma_{\text{lg}}\cos\theta =\gamma_{\text{sg}}-\gamma_{\text{sl}}$$

where $\gamma_{\text{lg}}$, $\gamma_{\text{sg}}$, and $\gamma_{\text{sl}}$ represent the liquid–gas, solid–gas, and solid–liquid interfacial tensions, respectively.

**FIGURE 2:**
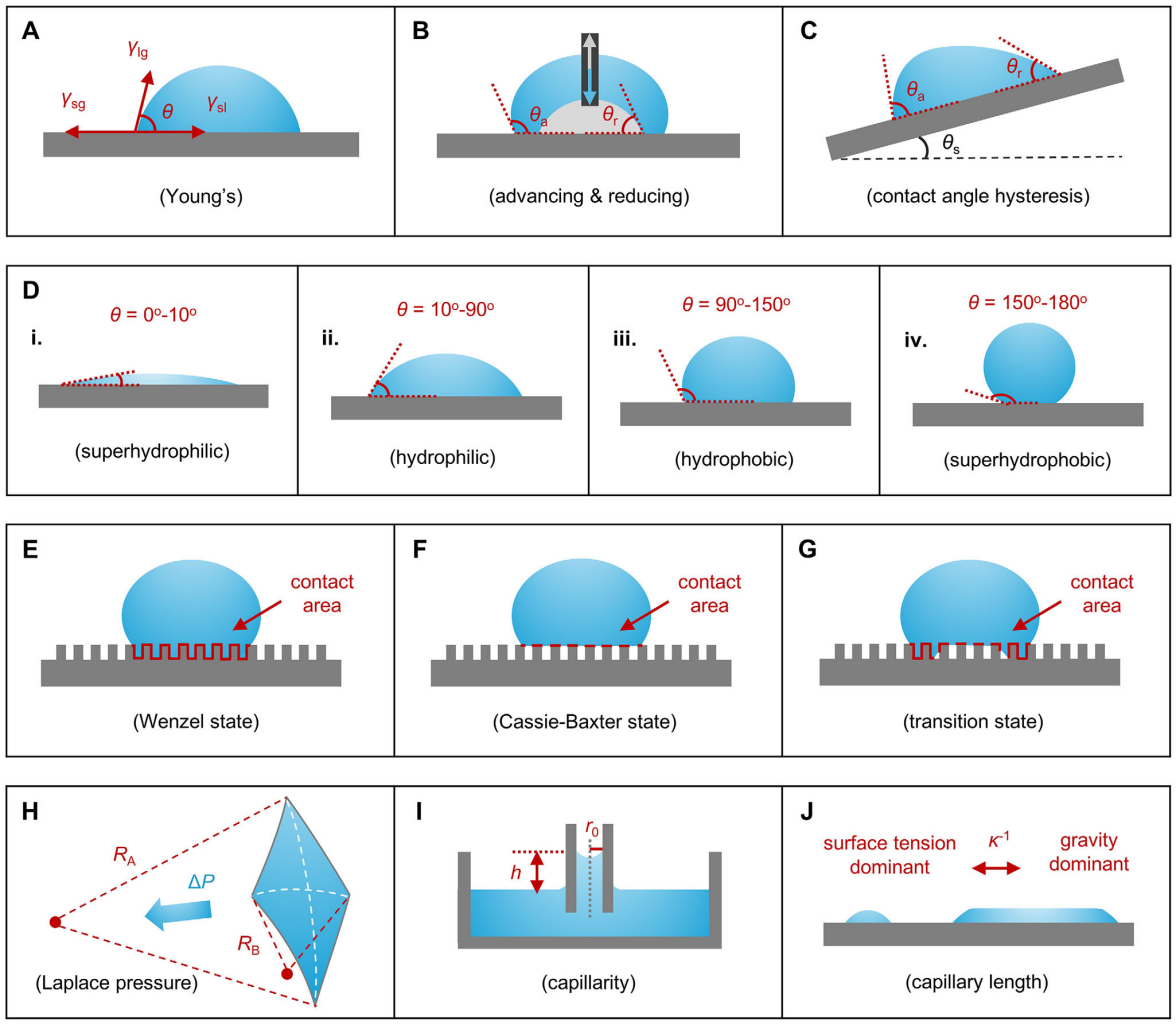
Fundamentals of wetting phenomena and liquid manipulation mechanisms. (A) Young's equation. (B) Advancing and receding contact angles. (C) Contact angle hysteresis and sliding angle. (D) Typical wetting regimes: (i) superhydrophilic, (ii) hydrophilic, (iii) hydrophobic, (iv) superhydrophobic. (E) Wenzel wetting state. (F) Cassie–Baxter wetting state. (G) Transition between the Wenzel and Cassie–Baxter states. (H) Laplace pressure across the curved interface. (I) Capillary rise in a tube. (J) Capillary length delineating the dominance of surface tension versus gravity.

However, this ideal equilibrium is rarely observed in practice. Real surfaces, with inherent roughness and chemical heterogeneity, cause the three‐phase contact line to resist motion. As shown in Figure 2B, during droplet expansion (illustrated in blue), θ reaches a maximum before the contact line advances, termed the advancing contact angle ($\theta_{a}$); during retracting (illustrated in gray), θ reaches a minimum value just before it recedes, known as the receding contact angle ($\theta_{r}$). Their difference (Δθ = $\theta_{a}$ ‐ $\theta_{r}$), called contact angle hysteresis (CAH) [85], can quantify the energy dissipation and resistance to droplet motion on surfaces. The smaller CAH corresponds to easier droplet roll‐off, which facilitates self‐cleaning; the larger CAH necessitates stronger pinning, a requirement for processes like retention and coating.

A more direct measure of droplet mobility on an inclined surface is the sliding angle ($\theta_{s}$) (Figure 2C). This is defined as the minimum tilt angle of a surface required to initiate the movement of a droplet of a defined volume. Furmidge has proposed an equation for calculating $\theta_{s}$ [86],

(2) $$\frac{mg\sin\theta_{s}}{w}=\gamma \left(\cos\theta_{r}-\cos\theta_{a}\right)$$

where γ is the liquid surface tension, *m* represents the droplet's weigh, *g* is the gravitational acceleration, θ denotes the liquid surface tension, and *w* indicates the width of the droplet's contact circle.

Engineered through chemistry and texture, surfaces can exhibit diverse wetting behaviors that are commonly classified into four states based on their apparent CAs. These include superhydrophilic ($0^{\circ }-10^{\circ }$), where the liquid spreads into a film (Figure 2D–i); hydrophilic ($10^{\circ }-90^{\circ }$), where the liquid wets the surface favorably (Figure 2D‐ii); hydrophobic ($90^{\circ }-150^{\circ }$), where the liquid beads up (Figure 2D‐iii); and superhydrophobic ($150^{\circ }-180^{\circ }$), where droplets are nearly spherical and highly mobile (Figure 2D‐iv).

To understand the origin of superwetting states, two key models for rough surfaces are crucial. The first is the Wenzel state [30], where the liquid penetrates surface textures and results in fully wetted contact areas (Figure 2E). Wenzel's equation incorporates the surface roughness factor, *r*, defined as the ratio of the actual surface area to the apparent area, and the apparent CA ($\theta_{W}$) is described by,

(3) $$\cos\theta_{W}=r\cos\theta$$

In this state, rough surfaces (r>1) will amplify the intrinsic wettability, that is, hydrophilic surfaces become more hydrophilic, and hydrophobic surfaces grow increasingly hydrophobic. However, for surfaces with extreme roughness, this model will yield mathematically invalid results $\cos\theta_{W}>1$, indicating that other parameters also affect the apparent CA. In such instances, the Cassie–Baxter model is applicable [31], as it accounts for chemical heterogeneity and composite interfaces. Here, the droplet cannot occupy all the voids on the rough surface, thus leading to the entrapment of air pockets beneath it, classified as non‐wetting (Figure 2F). Cassie and Baxter formulated the equation for the apparent CA ($\cos\theta_{\text{CB}}$) as follows:

(4) $$\cos\theta_{\text{CB}}=f_{\text{sl}}\left(\cos\theta +1\right)-1$$

where $f_{\text{sl}}$ is the area fraction of the solid surface in contact with the liquid. The Cassie–Baxter state is often metastable. Under external stimuli such as pressure, vibration, or an electric field, the liquid can infiltrate the textures and trigger a Cassie‐to‐Wenzel wetting transition (Figure 2G) [87]. This transition is crucial for designing surfaces with switchable adhesion in active liquid control systems.

Beyond static wetting, the spontaneous liquid propulsion is influenced by surface tension and capillary forces. A key factor is the Laplace pressure (Figure 2H), which arises from the curvature of the liquid interface. The pressure difference (ΔP) across this curved interface is given by the Young‐Laplace equation:

(5) $$\Delta P=-\gamma \left(\frac{1}{R_{A}}+\frac{1}{R_{B}}\right)$$

where $R_{A}$ and $R_{B}$ are the principal radii of curvature. ΔP is inversely proportional to the curvature radius, and compels the liquid to flow from regions of lower to higher curvature.

This pressure difference is the same force that drives capillarity, the ability of a liquid to spontaneously flow into narrow tubes or channels without external assistance, even against opposing forces like gravity. The height (*h*) to which a liquid of density ρ will rise in a vertical capillary tube of radius $r_{0}$ is given by Jurin's law [24], derived from balancing the capillary pressure with the hydrostatic pressure (Figure 2I)

(6) $$h=\frac{2\gamma \cos\theta }{\rho gr_{0}}$$

The extent of capillary action, however, is not unlimited and ultimately constrained by gravity. The relative importance of capillary forces over gravitational forces is defined by a characteristic scale known as the capillary length ($\kappa^{-1}$)

(7) $$\kappa^{-1}=\sqrt{\frac{\gamma }{\Delta \rho g}}$$

where Δρ is the mass density difference between the bottom and top phase. This length represents the maximum scale at which surface tension can maintain a static liquid body against gravity without significant collapse or flattening (Figure 2J). For common molecular fluids, the interfacial tension and density difference are typically on the order of 10–100 mN/m and 0.1–1 g/mL, respectively. Thus, the capillary length for such systems is ∼3 mm, as exemplified by the capillary length of water in contact with glass. This scale is paramount in microfluidics and many biological processes, a domain where capillary forces dominate at the sub‐millimeter scale and enable precise, passive control over liquid transport.

These interfacial principles offer a basic framework for designing functional surfaces. The following sections examine how they are utilized in distinct liquid manipulation modalities: passive systems that rely on surface chemistry and structure (Section 3); active systems that employ external stimuli for dynamic control (Section 4); and hybrid systems that integrate both approaches (Section 5).

## 3 Passive Liquid Manipulation Surfaces: Lessons from Nature

Through billions of years of evolution, natural systems have developed diverse passive liquid manipulation strategies (corresponding to the branch 1 in Figure 1) based on their structural intricacy and/or chemical properties to achieve passive liquid control without external energy input [1, 88, 89]. Although many of these strategies emerged independently and rely on distinct mechanisms rather than a sequential evolution of principles, this section organizes them into a conceptual framework to improve readability and contextual understanding (Figure 3). We first introduce nature‐inspired surface models, from homogeneous surfaces (Section 3.1) governing wetting‐state regulation to heterogeneous surfaces (Section 3.2) utilizing interfacial asymmetry for directional transport. We then transition to engineered passive systems for discrete droplet dynamics (Section 3.3), where transient interfacial‐energy conversion gives rise to behaviors such as droplet bouncing and jumping. Finally, we explore passive liquid manipulation strategies spanning directional transport, multidirectional control, and liquid programming, where pre‐designed energy landscapes enable complex routing behaviors and multifunctional operations (Section 3.4).

**FIGURE 3:**
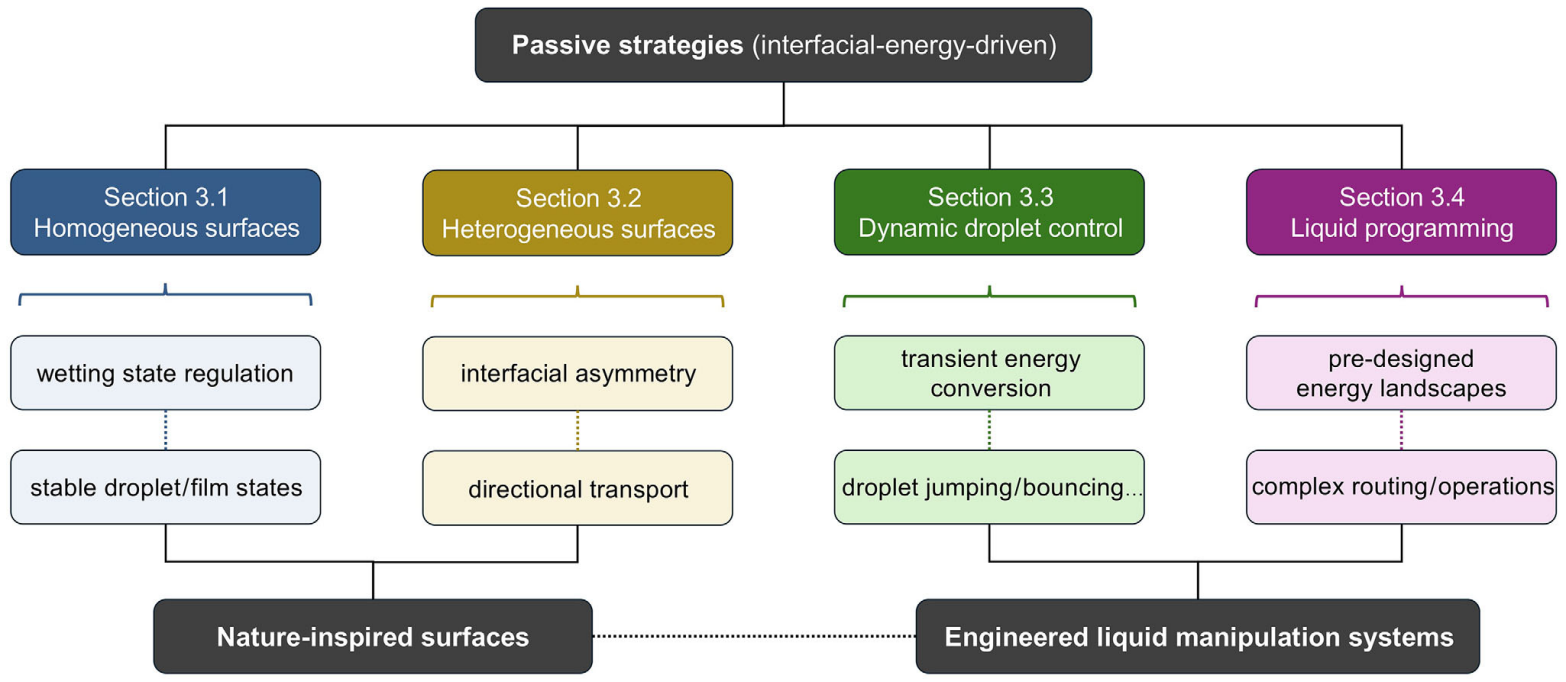
Organizational framework of passive liquid manipulation, from homogeneous and heterogeneous biomimetic surface models to engineered droplet dynamics and liquid programming.

### 3.1 Homogeneous Biological Surfaces

Homogeneous biological surfaces are characterized by uniform surface chemistry, where remarkable wettability mainly arises from hierarchical micro/nano‐scale physical structures. These structures regulate the morphology and stability of the three‐phase contact line, and enable extreme liquid‐repellent or liquid‐affinity properties. Figure 4 presents typical natural examples of superhydrophobicity, superhydrophilicity, and even superomniphobicity (repelling both water and oils) achieved through structural design.

**FIGURE 4:**
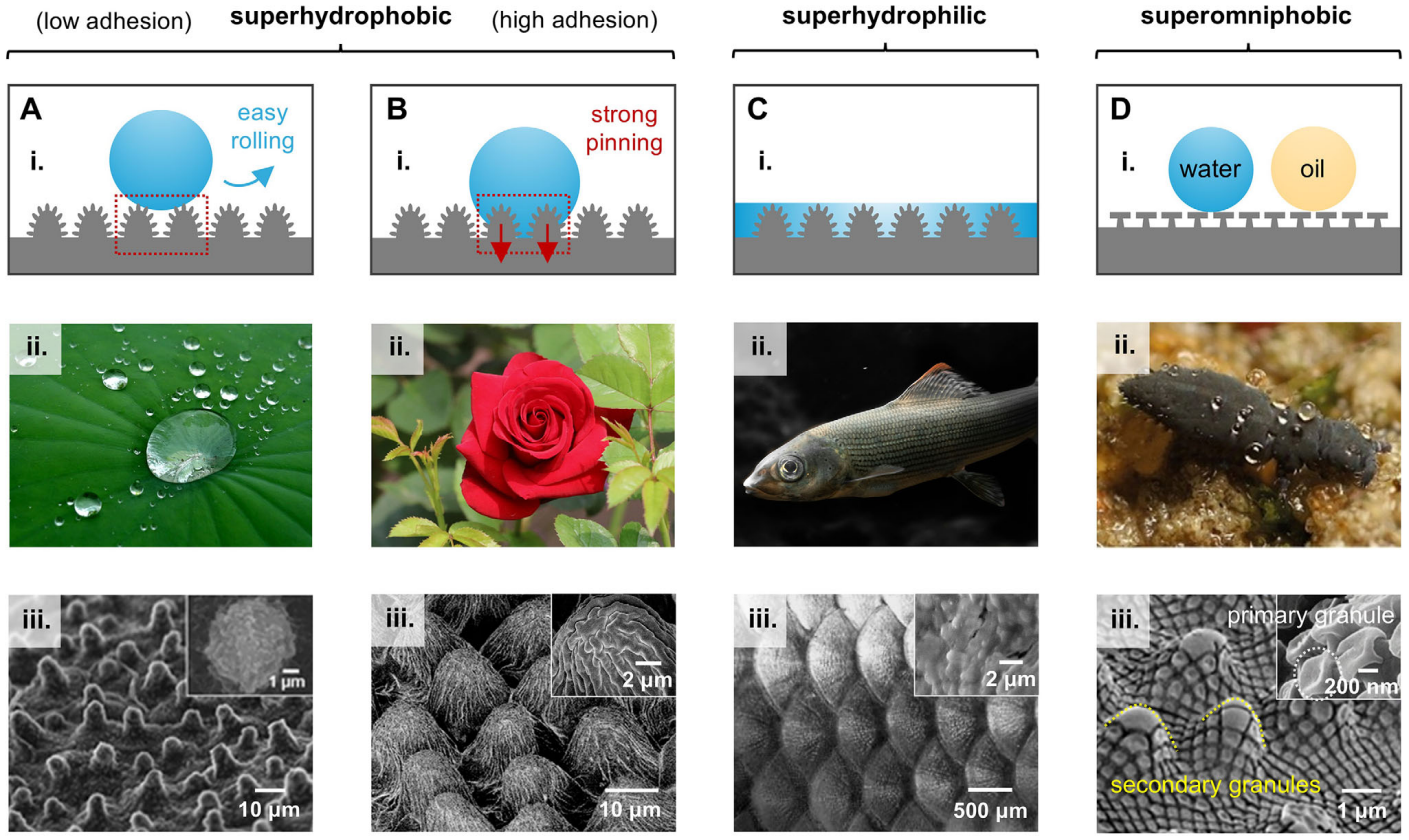
Representative homogeneous biological surfaces with superwetting properties. (A) Lotus leaf as a model for low‐adhesion superhydrophobicity: (i) schematic, (ii) photograph, and (iii) SEM images of its micro/nanostructures. Reproduced with permission [91]. Copyright 2008, Royal Society of Chemistry. B) Rose petal as a model for high‐adhesion superhydrophobicity: (i) schematic, (ii) photograph, and (iii) SEM images of its micro/nanostructures. Reproduced with permission [92]. Copyright 2008, American Chemical Society. C) Fish skin as a model for superhydrophilicity and underwater superoleophobicity: (i) schematic, (ii) photograph, and (iii) SEM images of its scale micro/nanostructures. Reproduced with permission [94]. Copyright 2009, Wiley‐VCH. D) Springtail cuticle as a model for superomniphobicity: (i) schematic, (ii) photograph, and (iii) SEM images of its hierarchical cuticular structures. Reproduced with permission [99]. Copyright 2018, AAAS.

The lotus leaf is a classic example of low‐adhesion superhydrophobicity (Figure 4A‐i–iii). Its hierarchical micro‐papillae and branch‐like nanostructures trap a stable air layer beneath the droplet, forming composite interfaces with high CA, low $\theta_{s}$, and easy roll‐off [32, 90, 91]. By contrast, the rose petal (Figure 4B‐i–iii) exhibits a high‐adhesion superhydrophobic state [92]. Although it also exhibits hierarchical micro/nano‐structures and high CA, partial liquid penetration into the structures increases solid–liquid interaction, resulting in strong adhesion and pronounced droplet pinning. Besides, superhydrophobicity also enables organisms such as water striders to walk on water via their superhydrophobic legs [93].

Fish scales exemplify superhydrophilic surfaces with underwater superoleophobicity (Figure 4C‐i, ii). As shown in the scanning electron microscope (SEM) images (Figure 4C‐iii), each scale comprises radially aligned micropapillae with nanoscale roughness. This hierarchical microstructure and its hydrophilic mucus coating enable the scales to trap a stable hydration layer when submerged and achieve underwater superoleophobicity. From a retrospective conceptual perspective, this example illustrates how liquid repellency can arise from stabilizing an immiscible liquid within surface structures [94]. A related but independently developed strategy is embodied in slippery liquid‐infused porous surfaces (SLIPSs) [95], where lubricating liquids are intentionally infused into porous textures to create repellent interfaces with excellent anti‐biofouling and abrasion resistance [96, 97].

Nature also provides solutions for repelling low‐surface‐tension liquids such as oils. A masterful case is the superomniphobic springtail cuticle [98], whose repellency arises from hierarchical mushroom‐shaped nanostructures with doubly reentrant geometry atop microscale grooves (Figure 4D‐i–iii) [99, 100]. These structures effectively suspend liquid interfaces and prevent infiltration by low‐surface‐tension liquids. The bioinspired concept of multiscale reentrant textures [73], from single to doubly reentrant and beyond [46, 101, 102, 103], has been fundamental in designing artificial superomniphobic surfaces. However, practical implementation requires not only extreme repellency but also mechanical durability and scalable fabrication. Representative studies have addressed this challenge using hierarchical micro/nano‐structures in which durable microscale frameworks protect fragile hydrophobic nanostructures from abrasion [104]. Besides the above structural approaches, chemical fabrication methods such as chemical vapor deposition, solution immersion, and electrodeposition also provide versatile routes for producing superhydrophobic surfaces [105], with the advantages of low cost, simple processing, and ease promotion.

### 3.2 Heterogeneous Biological Surfaces

This section examines heterogeneous biological surfaces that achieve directional liquid transport through structural and chemical anisotropy. By harnessing asymmetric interfacial forces, these natural systems passively steer liquids along predetermined paths. This approach provides a robust blueprint for the design of functional surfaces that direct liquid flow without external energy input.

Wettability gradients represent a simple yet effective strategy for liquid transport [106, 107]. The unbalanced surface tension generates a driving force ($F_{\text{gradient}}$) to propel droplets toward lower‐CA regions:

(8) $$F_{\text{gradient}}∼R_{0}\gamma \left(\cos\theta_{a}-\cos\theta_{r}\right)$$

where $R_{0}$ is the droplet radius. A classic example is the desert beetle [33], whose back is patterned with hydrophobic (wax‐coated) and hydrophilic (non‐waxy) regions (Figure 5A‐i, ii). This creates a surface energy gradient that directs condensed water droplets for efficient collection. A related principle governs droplet transport on stiffness‐gradient surfaces, where droplets spontaneously migrate toward softer, more wettable regions through surface energy minimization (Figure 5B‐i, ii). The droplet's surface tension deforms the soft underlying substrate, thus creats a gradient in the droplet's CAs. Despite being opposite to cell durotaxis (toward stiffer areas) [108], it demonstrates how to operate droplets without requiring external stimuli or topographical gradients [109].

**FIGURE 5:**
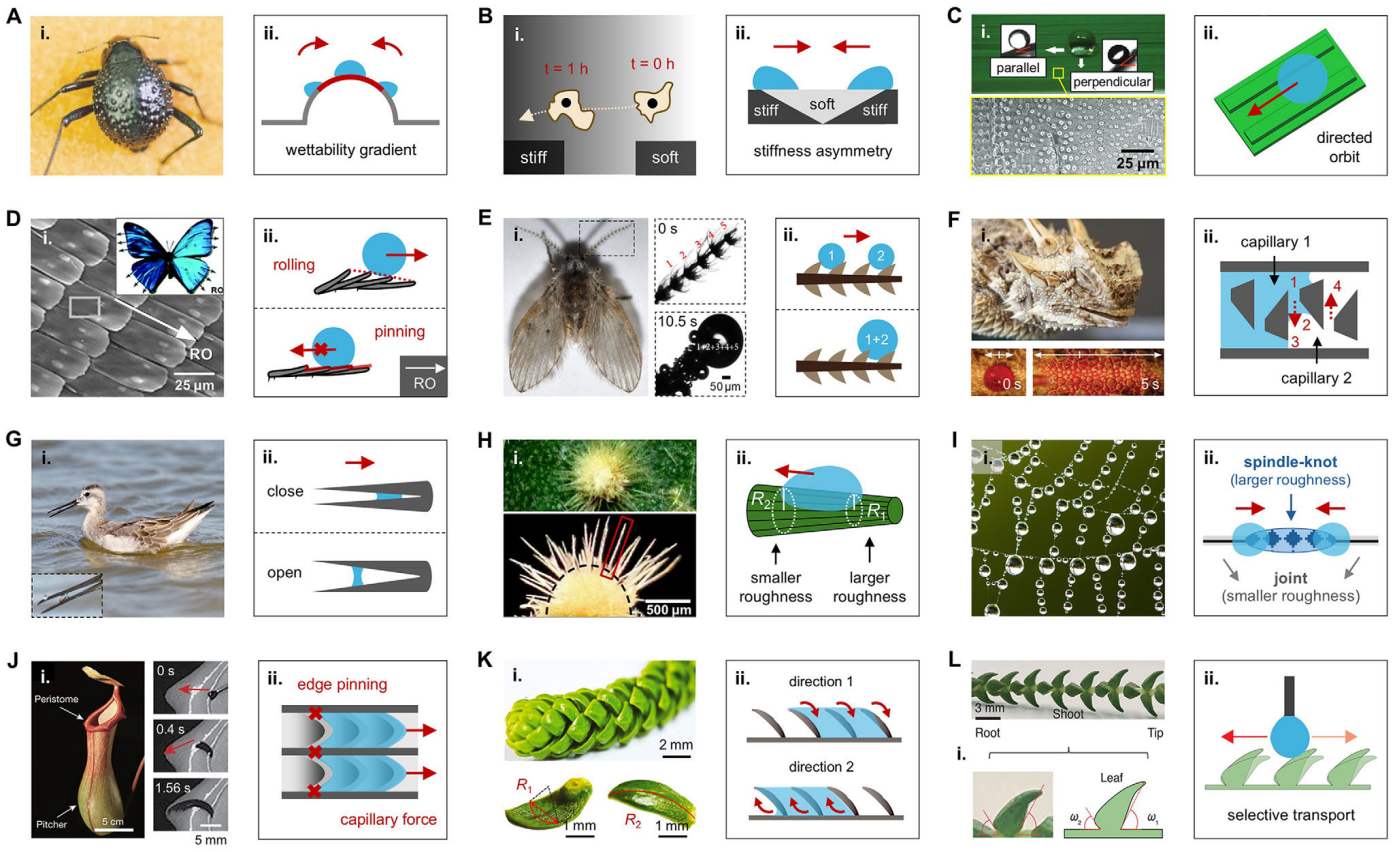
Liquid manipulation strategies on heterogeneous biological surfaces. (A) The desert beetle with a wettability gradient surface: (i) photograph of the beetle; (ii) schematic of droplet movement from hydrophobic to hydrophilic region (red line). Reproduced with permission [33]. Copyright 2001, Springer Nature. (B) Droplet durotaxis analogue: (i) cell durotaxis schematic; (ii) schematic of droplet migration from stiff to soft substrate. (C) Directional transport on rice leaves: (i) droplet motion and the surface SEM image; (ii) schematic of the directed droplet trajectory. Reproduced with permission [110]. Copyright 2013, Wiley‐VCH. (D) Directional droplet movement on a butterfly wing: (i) wing photograph and the SEM image; (ii) droplet pinning and rolling schematic. Reproduced with permission [111]. Copyright 2007, Royal Society of Chemistry. (E) Drain fly tentacle as a droplet rectifier: (i) images of droplet rectification; (ii) droplet transport schematic. Reproduced with permission [112]. Copyright 2020, AAAS. (F) Directional liquid transport on the Texas horned lizard skin: (i) photograph and directional liquid spreading; (ii) sequential depinning mechanism via liquid diode. Reproduced with permission [114]. Copyright 2015, Royal Society. (G) Shorebird beak drinking: (i) photograph; (ii) schematic of directional liquid movement during beak opening/closing. Reproduced with permission [35]. Copyright 2008, AAAS. (H) Water collection on a cactus spine: (i) macro‐ and close‐up views of the cactus spine; (ii) transport driven by Laplace pressure asymmetry and roughness gradient. Reproduced with permission [34]. Copyright 2012, Springer Nature. (I) Water collection on spider silk: (i) photograph; (ii) directional transport driven by combined Laplace pressure asymmetry and roughness‐mediated wettability gradients. Reproduced with permission [36]. Copyright 2010, Springer Nature. (J) Directional liquid transport on the pitcher plant peristome: (i) experimental image of liquid transport within 1.56 s; (ii) schematic of the edge‐pinning and capillary‐force mechanism. Reproduced with permission [37]. Copyright 2016, Springer Nature. (K) Bidirectional liquid transport on the *Araucaria* leaf: (i) the leaf's dual‐curvature structure; (ii) schematic of bidirectional liquid transport. Reproduced with permission [39]. Copyright 2021, AAAS. (L) Selective liquid transport on *Crassula muscosa* shoots: (i) photograph and structure schematic; (ii) selective transport via structural parameters. Reproduced with permission [40]. Copyright 2024, AAAS.

Anisotropic topography provides another powerful transport mechanism. Rice leaves possess aligned microgrooves and nanostructures that promote preferential spreading along the groove direction (Figure 5C‐i, ii) [110]. This longitudinal arrangement creates directional resistance: while liquid spreads more easily along the grooves, it encounters a higher energy barrier across them, resulting in anisotropic droplet sliding. Another strategy appears on butterfly wings. They employ radially oriented overlapping scales (Figure 5D‐i, ii), guiding droplets outward to maintain wing dryness and flight efficiency in humid conditions [111].

Certain organisms integrate multiple structural features to achieve directional droplet manipulation. The drain fly tentacle combines multiscale roughness, periodicity, and flexibility into tilted ratchet arrays of knots that function as a rectifier (Figure 5E‐i, ii) [112]. These arrays use capillary forces to propel condensed droplets directionally; repeated coalescence then transports droplets to the tip, thereby preventing liquid flooding. Likewise, the pine needle employs inclined Janus pillar arrays with a height gradient to direct droplet transport [113]. The asymmetric tip flips droplets from the flat to the curved side, which enables spontaneous, long‐distance self‐transport through continuous coalescence.

The Texas horned lizard shows a liquid diode based on interconnected capillary channels with varying widths (Figure 5F‐i, ii) [114]. This geometry promotes unidirectional flow through capillary action and overcomes liquid pinning via a sequential depinning mechanism. When liquid advances in one channel (direction 2), it merges with and depins a pinned front at a junction (point 3). This newly merged front advances further, depinning another stalled liquid (point 1) through an interconnecting channel (direction 4). This relay‐like process ensures continuous forward transport, while reverse flow is blocked at widening sections that lack the interconnections necessary for depinning.

Laplace pressure gradients generated by asymmetric structures offer a dynamic pumping strategy. A prominent example is the shorebird's beak [35], where rhythmic opening and closing generate curvature‐induced pressure gradients that pump liquid toward narrower regions while preventing backflow (Figure 5G‐i, ii). For a droplet with width *W*, length *L*, and distance from the beak base *X*), the resulting capillary force ($F_{c}$) can be derived as

(9) $$F_{c}∼\gamma \cos\theta \frac{WL}{X}$$

These gradients can be further combined with surface energy gradients to achieve robust directional liquid control. The cactus spine utilizes a conical geometry that synergizes Laplace pressure and wettability gradients to direct condensed fog toward its base (Figure 5H‐i, ii) [34]. The macroscopic taper first generates a Laplace pressure differential (higher at the tip, lower at the base) to drive liquid toward the wider end. The resultant pressure difference, $\Delta P_{\text{curvature}}$, expressed as,

(10) $$\Delta P_{\mathrm{curvature}}=-\int_{R_{1}}^{R_{2}}\frac{2\gamma }{{\left(R+R_{0}\right)}^{2}}\sin\alpha dz$$

where *R* is the local spine radius ($R_{1}$ and $R_{2}$: radii at the droplet's two sides), $R_{0}$ is the droplet radius, α is the spine half‐apex angle, and d*z* is the incremental radius along the spine. Meanwhile, microgrooves generate a wettability gradient that cooperates with the Laplace pressure gradient, while trichomes near the base act as capillary pumps to suppress backflow.

The spider silk provides another example of directional liquid transport driven by coupled Laplace pressure and wettability gradients [36]. Its periodic spindle knots and joints create alternating curvature and distinct wettability (Figure 5I‐i, ii), generating a Laplace pressure gradient that drives droplets toward the knots while enhanced roughness increases local hydrophilicity. This wettability gradient acts together with the Laplace pressure gradient to improve water collection efficiency. By tuning knot size and surface roughness, the direction and efficiency of droplet transport can be precisely controlled [115].

The pitcher plant peristome is another bioinspired strategy for rapid directional liquid transport [37]. High‐speed imaging reveals directional water motion in microchannels perpendicular to the peristome rim (Figure 5J‐i). This continuous transport arises from capillary‐driven flow within partially confined microcavities (Figure 5J‐ii). Sharp edges pin the liquid, while wedge‐shaped corners guide meniscus propagation and sequential cavity filling, thereby sustaining forward transport and suppressing backflow. This mechanism has inspired various continuous capillary transport systems [116, 117]. In addition, the pitcher plant's ability to lock lubricating liquid films within microtextures has also inspired the design of SLIPS [95, 118].

While many asymmetric surfaces exhibit predetermined unidirectional transport, some biological systems demonstrate programmable liquid manipulation. For example, the *Araucaria* leaf [39], with periodically arranged three‐dimensional (3D) ratchet structures (Figure 5K‐i, ii), creates distinct spreading pathways for liquids with different surface tensions. Similarly, the shoot surface of *Crassula muscosa* achieves bidirectional transport through asymmetric reentrant leaves with varying inclination angles, enabling the same liquid to move along selectable directions (Figure 5L‐i, ii) [40]. These natural mechanisms provide a biomimetic foundation for recent engineered surfaces with programmable liquid transport [47, 71, 119, 120, 121].

Nature employs both homogeneous superwetting surfaces and heterogeneous anisotropic structures to manipulate liquids with remarkable precision and efficiency. These strategies provide fundamental design principles for engineered surfaces capable of directional, adaptive, and programmable liquid transport.

### 3.3 Passive Engineered Surfaces for Discrete Droplet Control

Inspired by hierarchical and heterogeneous designs in biological systems, various engineered surfaces have been developed for liquid control. This section focuses on discrete droplet dynamics on passive surfaces, including impact‐induced bouncing, directional rebound, rotation, and spontaneous jumping. These phenomena involve localized regulation of individual droplet behaviors through transient interfacial energy conversion rather than sustained liquid transport.

Droplet bouncing is a fundamental interfacial phenomenon with important applications in heat transfer, anti‐icing, and self‐cleaning [122, 123, 124, 125, 126]. Its key optimization metric is the contact time [127, 128], defined as the duration of droplet‐surface contact during impact. Minimizing contact time is crucial to suppress ice formation and improve thermal exchange efficiency. In this context, a pancake bouncing mode has been discovered on engineered hierarchical superhydrophobic surfaces composed of tapered posts with nanoscale textures (Figure 6A‐i) [129]. At low impact velocity, where Weber number (*We*) is equal to 7.1, droplets undergo classic complete rebound with full retraction before detachment (Figure 6A‐ii) [130, 131]. Yet, at *We* = 14.1, droplets detach immediately after maximum spreading in a flattened pancake shape (Figure 6A‐ii), reducing contact time from ∼16.5 ms to ∼3.4 ms. This behavior originates from rapid capillary‐energy release enabled by the tapered micro/nano‐structures. Similarly, *Echevaria* leaves achieve shortened contact time (∼40%) via asymmetric convex/concave architectures that induce preferential fluid pumping during impact [132]. These studies highlight the critical role of surface morphology in regulating droplet impact dynamics.

**FIGURE 6:**
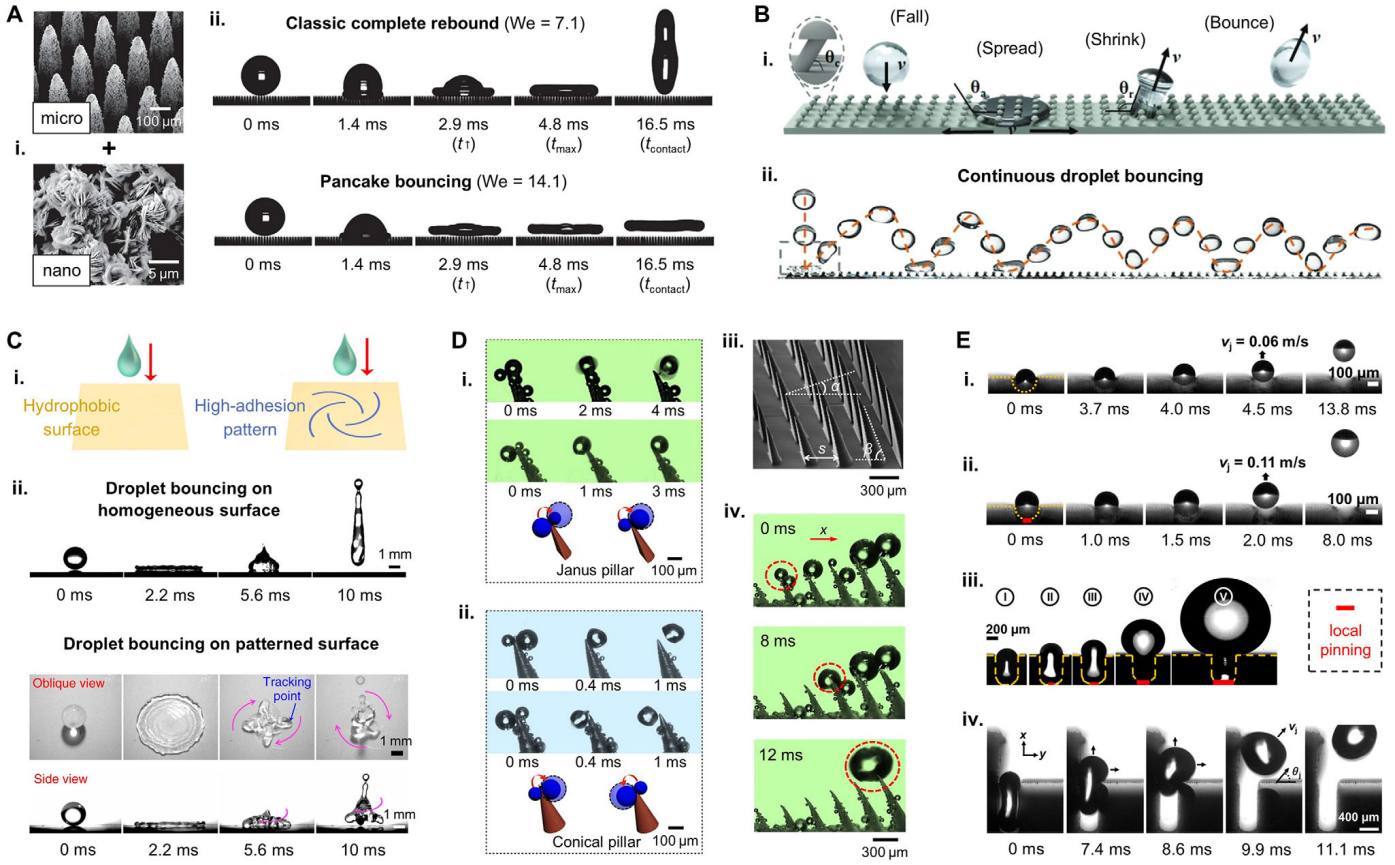
Passive engineered surfaces for discrete droplet control. (A) Anomalous droplet bouncing on hierarchical micro/nano‐structured surfaces: (i) SEM image of the surface morphology; (ii) comparison of classic rebound and pancake bouncing. Reproduced with permission [129]. Copyright 2014, Springer Nature. (B) Directional bouncing on inclined mushroom‐like microarrays: (i) schematic of a single bounce; (ii) experimental image of consecutive bounces. Reproduced with permission [133]. Copyright 2024, Wiley‐VCH. (C) Programmable droplet dynamics on hydrophobic surfaces with high‐adhesion spiral patterns: (i) schematics of surfaces without/with pattern; (ii) bouncing behavior on homogeneous and patterned hydrophobic surfaces, showing strong droplet rotation on the latter. Reproduced with permission [136]. Copyright 2019, Springer Nature. (D) Directional droplet self‐propulsion on pine‐needle‐inspired micropillar arrays: (i, ii) tip‐induced flipping on Janus pillars versus random transport on conical pillars; (iii) SEM image of the microarray; (iv) experimental images of continuous directional droplet transport. Reproduced with permission [113]. Copyright 2020, AAAS. E) Controlled single‐droplet jumping via groove‐based superhydrophobic surfaces: (i) spontaneous high‐performance droplet jumping; (ii, iii) velocity enhancement and volume‐regulated jumping via bottom pinning; (iv) directional droplet jumping achieved by groove designs with single‐side walls. Reproduced with permission [142]. Copyright 2020, American Chemical Society.

Beyond reducing contact time, surface morphology can steer droplet bouncing directions. Bioinspired mushroom‐like microarrays achieve this through geometric asymmetry [133]. After hydrophobic modification, these microstructures sustain continuous directional droplet bouncing (Figure 6B‐i, ii), with programmable trajectories and anti‐gravity transport capabilities. Such control offers potential applications in self‐cleaning and rainfall energy harvesting. Related strategies include sunflower‐inspired superhydrophobic micro‐cone arrays for precise bouncing regulation [134] and asymmetric interfacial structures for efficient droplet removal [135]. These studies showcase how designed surface asymmetry can form the basis of intelligent droplet manipulation systems.

Apart from directional control, another promising strategy for programming droplet dynamics is to convert translational motion into rapid rotation. By fabricating high‐adhesion spiral architectures on hydrophobic substrates (Figure 6C‐i), researchers have achieved controlled droplet rotation exceeding 7300 revolutions per minute [136], leading to innovative dynamic bouncing behaviors distinct from those on homogeneous surfaces (Figure 6C‐ii). This wettability patterning generates asymmetric pinning forces, which interact with the droplet's excess surface energy during spreading. The resulting synergy then converts linear momentum into sustained rotation via engineered adhesion gradients to establish new paradigms for programming droplet dynamics [137, 138].

In contrast to the impact‐driven bouncing discussed above, another passive mode of droplet regulation relies on spontaneous jumping rather than rebound from incoming momentum. Whereas bouncing is triggered by droplet impact, coalescence‐induced jumping arises from the release of interfacial energy during droplet growth or merging. Due to its importance in natural and engineering processes ranging from self‐cleaning and anti‐fogging to condensation heat transfer, coalescence‐induced jumping on superhydrophobic surfaces has emerged as one of the most efficient passive droplet shedding mechanisms [139, 140, 141]. During droplet coalescence, the reduction in liquid–air interfacial area releases excess surface energy, which is converted into kinetic motion by the non‐wetting substrate, leading to spontaneous droplet ejection.

To achieve directional transport from coalescence‐induced jumping, inspired by the structure of *Sabina chinensis* needles [113], engineered Janus micropillar surfaces induce tip‐driven droplet flipping that directs coalescing droplets toward the curved side (Figure 6D‐i), unlike conical pillars that produce non‐directional transport (Figure 6D‐ii). By combining height gradient (α), tilt angle (β), and pillar spacing (*s*) (Figure 6D‐iii), these surfaces achieve long‐range directional droplet transport (Figure 6D‐iv), enabling efficient fog harvesting and water concentration. Beyond directional control, another challenge of coalescence‐induced jumping is limited energy‐conversion efficiency and jumping velocity. Groove‐based superhydrophobic surfaces address this by confining droplet growth and regulating Laplace‐pressure release, enabling single‐droplet jumping (Figure 6E‐i) [142]. Confining droplet growth within optimized grooves enhances jumping velocity and energy‐conversion efficiency. The introduction of local surface pinning, where surface defects act as high‐energy anchoring points, can greatly enhance jumping velocity (Figure 6E‐ii). When droplets encounter these pinning sites, a larger volume is required to generate sufficient Laplace pressure to overcome the pinning forces (Figure 6E‐iii). Moreover, engineered groove geometries enable programmable jumping trajectories with tunable ejection angles (Figure 6E‐iv).

Droplet dynamics is also affected by liquid properties and environmental conditions. Distinct bouncing behaviors have been reported for viscous [143], hollow [144], and particle‐laden droplets [145] on superhydrophobic surfaces. Environmental factors further enrich droplet dynamics, including spontaneous trampolining under reduced pressure [146], Leidenfrost levitation at elevated temperatures [147], and electrically induced active droplet jumping [148]. These findings underscore the coupled roles of surface design, liquid properties, and external conditions in enabling precise droplet manipulation.

### 3.4 Passive Engineered Surfaces for Directional Control and Liquid Programming

Complementing the discrete droplet dynamics discussed in Section 3.3, this section focuses on passive engineered surfaces for sustained directional control and liquid programming, including long‐range transport, multidirectional routing, capture/release, and programmable fluidic operations.

A typical example is droplet transport driven by engineered surface energy gradients [53]. A diffusion‐controlled vapor treatment creates a continuous wettability gradient on a silicon wafer ($\theta_{A}<\theta_{B}$), with the water contact angle decreasing from $97^{o}$ to $25^{o}$ over 1 cm (Figure 7A‐i). This gradient induces an asymmetric surface tension along the droplet's contact line, and enables 1–2 μL droplets to migrate uphill at 1–2 mm/s on a $15^{o}$ slope (Figure 7A‐ii). Critical to this motion is the minimal CAH ($\leq 10^{o}$), which almost eliminates resistive pinning forces. This work establishes the principle of passive liquid manipulation enabled by precise surface chemistry [54, 149].

**FIGURE 7:**
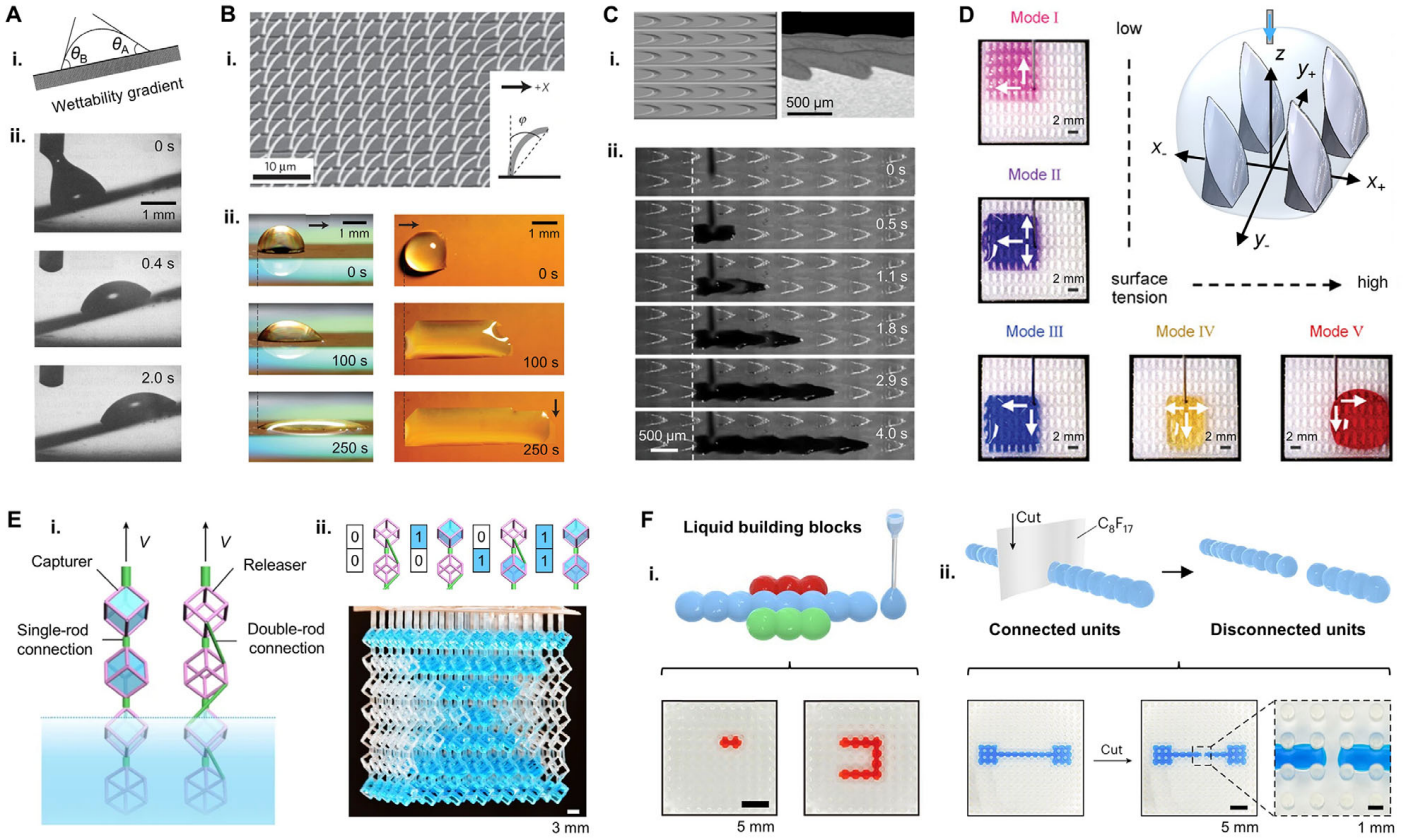
Directional control and liquid programming on passive engineered surfaces. (A) Droplet uphill transport on a chemically modified wettability‐gradient surface: (i) schematic; (ii) uphill spreading experiment. Reproduced with permission [53]. Copyright 1992, AAAS. (B) Directional liquid spreading on tilted nanostructured arrays: (i) SEM image of the nanopillar surface; (ii) experimental snapshots (side and top views) of directional transport over 250 s. Reproduced with permission [45]. Copyright 2010, Springer Nature. (C) Robust directional liquid transport on a pitcher‐plant‐inspired engineered surface: (i) SEM image of the biomimetic surface; (ii) experimental sequence of directional transport within 4 s. Reproduced with permission [117]. Copyright 2016, Wiley‐VCH. (D) Multidirectional transport modes on surfaces with multi‐asymmetric structural features. Reproduced with permission [119]. Copyright 2024, Wiley‐VCH. (E) Controllable liquid capture and release using connected polyhedral frames: (i) function of differently connected rods as capturers and releasers; (ii) programmed 3D liquid patterning experiment. Reproduced with permission [176]. Copyright 2024, Springer Nature. (F) Modular construction of liquid devices using droplets as building blocks: (i) schematic and experimental images of the building method; (ii) schematic and experimental images of the block reconfiguration. Reproduced with permission [178]. Copyright 2024, Springer Nature.

Structured surfaces with heterogeneous properties offer an alternative pathway for directional liquid control. Researchers have developed inclined nanostructures (bending angle: φ) that enable unidirectional liquid spreading (Figure 7B‐i), where liquid propagates along a single preferred direction while pinning in the opposite direction (Figure 7B‐ii) [45]. Directional spreading is governed by the degree of nanostructural asymmetry, the height‐to‐spacing ratio, and the intrinsic contact angle, demonstrating how engineered nanostructures can precisely regulate wetting and liquid transport.

Engineered surfaces inspired by heterogeneous natural architectures (Figure 5) have been realized through physical structuring, chemical patterning, or their combination, yielding various nature‐inspired configurations [150, 151, 152, 153, 154, 155, 156, 157, 158, 159, 160, 161, 162, 163, 164, 165, 166, 167, 168, 169, 170, 171]. Among these, the peristome‐inspired surface derived from *Nepenthes alata* is a refined system, which enables rapid, robust, and long‐range directional liquid transport without dependence on chemical modification. Researchers have fabricated these peristome‐mimicking surfaces and revealed the unidirectional transport mechanism (Figure 7C‐i) [117]. These surfaces exhibit an overflow‐controlled liquid transport phenomenon, where liquids with varying surface tensions and viscosities propagate in a single preferred direction while pinning in others (Figure 7C‐ii). Similarly, the trichome‐covered operculum of *Sarracenia* achieves ultrafast directional water transport through hierarchical microchannels that sustain lubricating liquid films for prey capture [38].

Beyond unidirectional transport, some plants hold 3D capillary ratchet structures enabling programmable multidirectional liquid control [39]. Inspired by this, engineered surfaces with ordered 3D asymmetric units achieve in‐situ customized multidirectional spreading, categorized as modes I–V (Figure 7D) [119]. This capability originates from distributed multi‐curvature structures that generate distinct Laplace‐pressure gradients for liquids with different wettabilities. Similar liquid control is frequently achieved on surfaces that integrate multiscale features [120, 172, 173, 174], such as curvature, tilt, and micro‐textures, which synergistically generate complex interfacial forces to regulate anomalous transport behaviors.

Passive liquid manipulation has also evolved from directional transport toward programmable fluidic systems. Researchers have proposed cellular fluidics [175], a platform that uses 3D‐printed unit cells to guide multiphase flows and reactions through programmable geometries. Related approaches use connected polyhedral frames to regulate liquid continuity between structural units [176, 177]. By tailoring connection geometries (Figure 7E‐i), these architectures enable programmable liquid capture/release, spatiotemporal transport control, and massively parallel operations (Figure 7E‐ii). Modular droplet‐based assembly strategies further extend liquid programmability by enabling rapid construction and reconfiguration of functional liquid devices on structured substrates (Figure 7F‐i, ii) [178, 179]. These approaches support versatile applications in fluidic devices, microreactors, and programmable multiphase systems.

Section 3 has traced the evolution from biological inspiration to engineered passive systems for liquid manipulation. These approaches exploit intrinsic interfacial energy through wettability gradients and asymmetric surface architectures to achieve diverse liquid operations with exceptional energy efficiency. However, their functionality is constrained once the surface architecture is fixed. The resulting liquid behaviors are predetermined by the static interfacial energy landscape and cannot adapt to changing tasks or environmental conditions. As liquid manipulation systems move toward more complex operations, such as programmable routing, dynamic droplet sorting, and multi‐step fluidic processing, these limitations become increasingly evident. To address these limitations, external‐field actuation has been introduced. In the following section, we examine active liquid manipulation methods based on external stimuli that provide the flexibility and real‐time controllability lacking in passive systems.

## 4 Active Liquid Manipulation: Stimuli‐Responsive Dynamics

Active liquid manipulation leverages dynamic external fields to induce on‐demand and often reversible changes in surface properties or liquid behaviors. Corresponding to the branch 2 in Figure 1, these strategies encompass representative control modalities based on thermal, electric, magnetic, optical, and acoustic stimuli, rather than a sequential technological progression. By converting external energy into morphological reconfiguration or interfacial‐energy modulation (i.e., adjustment of interfacial properties or responses), active systems enable real‐time and adaptive liquid control. This section examines the mechanisms, functionalities, and strengths/limitations of these active strategies. We focus on interfacial liquid manipulation strategies, and broader reviews of field‐driven microfluidic systems can refer to the existing literature [180, 181, 182, 183, 184, 185].

### 4.1 Thermal‐Driven Liquid Manipulation

While temperature‐mediated phase transitions are ubiquitous in our daily life, the role of temperature in governing liquid behavior extends further. A prime example is the Leidenfrost effect [147], where droplets levitate on a vapor layer above the boiling point, significantly reducing evaporation (Figure 8A). This phenomenon has attracted broad interest for applications in heat transfer, fluid transport, drag reduction, and green chemistry [186]. From the wetting mechanism perspective, a key principle is the temperature dependence of surface tension [187]. Because surface tension decreases with temperature, thermal gradients generate surface tension gradients that drive Marangoni flow from hotter (low‐tension) to colder (high‐tension) regions [180, 188]. Unlike wettability‐gradient‐driven transport described by Equation (8), thermocapillary transport is governed by internal Marangoni flow and can drive liquids toward regions with larger apparent CA (Figure 8B) [189].

**FIGURE 8:**
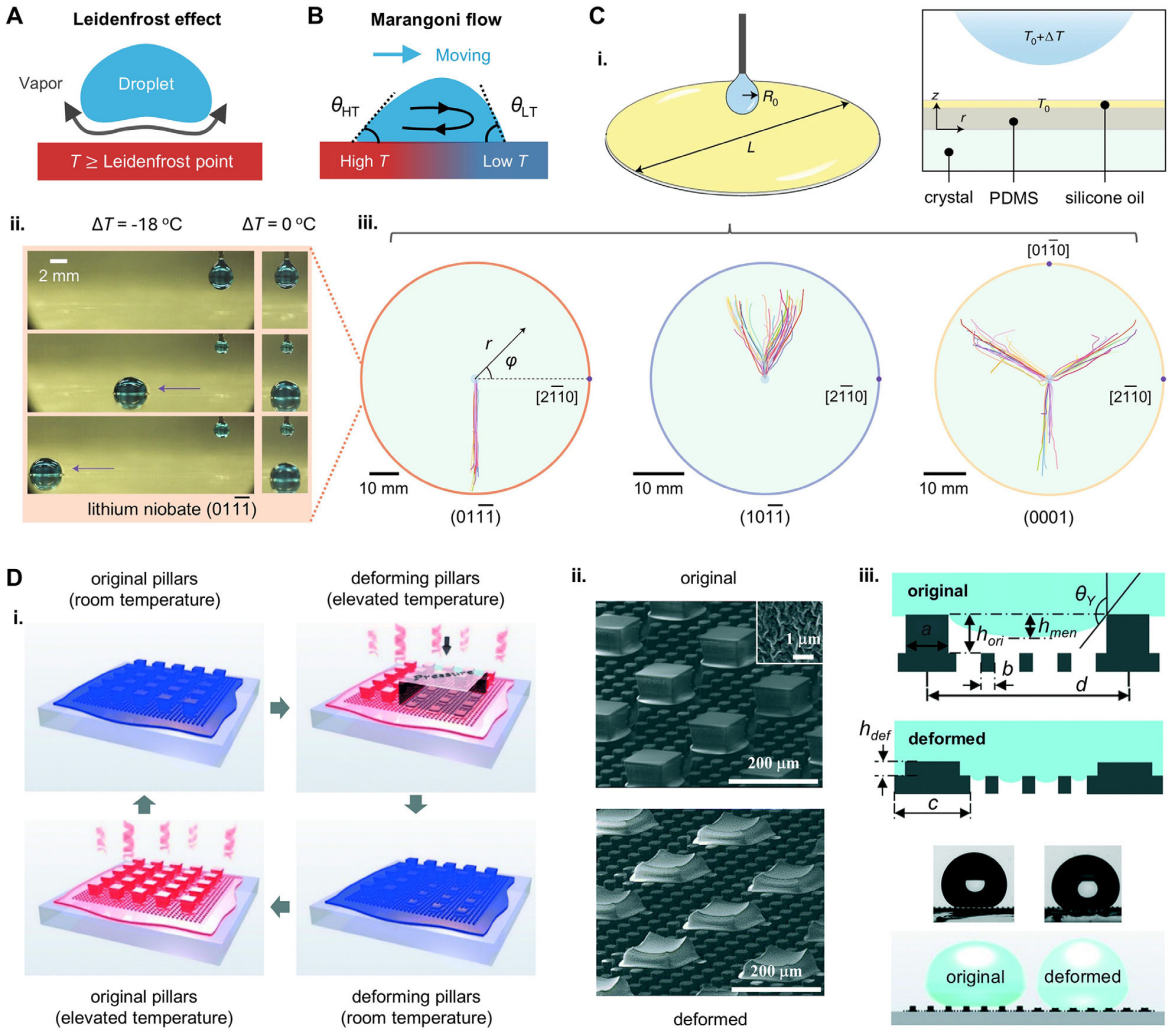
Active liquid control via thermal fields. (A) Schematic of the Leidenfrost effect. (B) Marangoni‐flow‐driven droplet motion under a thermal gradient. (C) Thermally induced droplet self‐propulsion on anisotropic ${\text{LiNbO}}_{3}$ crystals: (i) schematic of crystal surfaces coated with PDMS and silicone oil layers; (ii) experimental comparison of droplet motion (with temperature difference) versus no motion (without temperature difference); (iii) unidirectional, bifurcated, or trifurcated trajectories driven by material‐anisotropy‐induced electric potentials. Reproduced with permission [190]. Copyright 2021, Springer Nature. (D) Droplet manipulation on SMP surfaces: (i) mechanism of surface morphology change with temperature; (ii) SEM images of original and deformed SMP surfaces; (iii) different wetting states caused by solid–liquid‐as interface changes on original versus deformed surfaces. Reproduced with permission [195]. Copyright 2017, Royal Society of Chemistry.

Distinct from conventional thermal‐driven liquid transport, thermoelastic–piezoelectric coupling in anisotropic crystals such as ${\text{LiNbO}}_{3}$ enables droplet self‐propulsion without engineered surface asymmetry [190]. This counterintuitive motion arises on specially prepared crystal surfaces coated with polydimethylsiloxane (PDMS) and silicone oil layers (Figure 8C‐i), where a temperature difference ΔT (around –18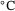) between the droplet and substrate is transformed by the material's intrinsic anisotropy into complex electric potentials. These potentials guide droplets along defined paths, including unidirectional, bifurcated, or trifurcated trajectories (Figure 8C‐ii, iii). Yet, the self‐propulsion ceases at ΔT = 0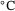, highlighting the essential role of thermal effect.

Thermal actuation can further be integrated with smart materials to regulate surface morphologies. For example, poly(*N*‐isopropylacrylamide) (PNIPAM) undergoes a reversible conformational transition across its lower critical solution temperature (LCST, $∼32^{\circ }C$). Below the LCST, the hydrated polymer chains are extended and hydrophilic; while above the LCST, they collapse into a globular, hydrophobic state. This transition enables reversible wettability control for droplet transport and adhesion regulation [67, 191]. In addition, the associated volumetric deformation makes PNIPAM attractive for soft actuators [192, 193]. By incorporating photothermal materials, light can induce localized heating for structural deformation, and further directional liquid transport through coupled wettability and Laplace pressure gradients [194].

Shape memory polymers (SMPs) offer another thermally responsive strategy with programmable structural reconfiguration [195]. Researchers have developed a structured SMP surface with tunable wettability for droplet manipulation. It comprises an array of textured SMP pillars capable of reversible morphological switching between permanent and temporary shapes via thermomechanical programming (Figure 8D‐i). The original state exhibits a higher apparent CA than the deformed state (Figure 8D‐ii, iii). Further, local thermal activation induces asymmetric shape recovery and produces wettability gradients that, together with Marangoni effects, direct droplet motion.

Thermal fields provide a multifaceted liquid manipulation toolkit. Although thermal strategies may face limitations such as slow response, high energy consumption, and limited compatibility with temperature‐sensitive systems, they remain attractive for applications in thermal management, soft robotics, and programmable microfluidics.

### 4.2 Electric‐Driven Liquid Manipulation

Electric fields provide versatile platforms for rapid, reversible, and tunable liquid manipulation through electrohydrodynamic effects [196]. Supported by innovative electrode architectures and field modulation strategies, current studies have introduced electric‐driven techniques such as electrowetting‐on‐dielectric (EWOD) actuation, dielectrophoresis (DEP), electrostatic attraction/repulsion, and surface charge density gradients. This section explores their fundamental principles and shows the applicability in digital microfluidics, lab‐on‐a‐chip systems, and adaptive microdevices.

Electric‐field‐driven liquid manipulation has evolved from electrowetting to digital microfluidics (DMF). Electrowetting was first reported by Lippmann in 1875 [197], while Berge later introduced EWOD by incorporating a dielectric layer to suppress electrolysis and control droplets (Figure 9A‐i) [56]. By programming voltage sequences across electrode arrays, EWOD enables droplet generation, splitting, merging, and transport, forming the basis of DMF (Figure 9A‐ii, iii) [57]. This development has led to automated commercial platforms and established DMF as a major application of electric‐driven liquid manipulation [198, 199, 200].

**FIGURE 9:**
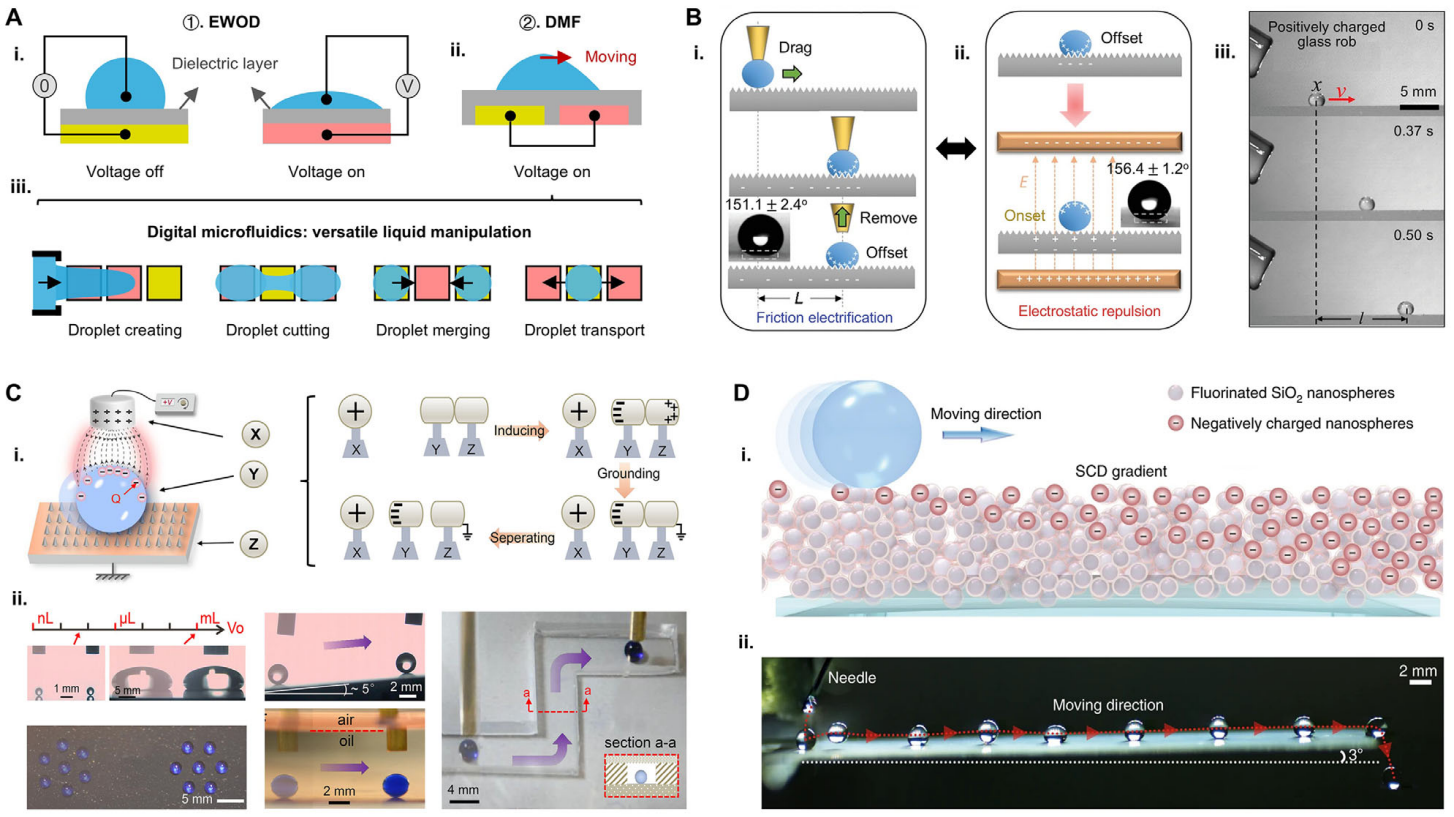
Droplet manipulation driven by electrical fields. (A) Digital microfluidics: (i) schematic of the EWOD principle that modulates wettability; (ii) droplet transport driven by neighboring electrodes; (iii) expanded DMF‐based droplet manipulation, including creation, cutting, merging, and transport. (B) Droplet manipulation via electrostatic forces: (i) charging process and CA modulation; (ii) CA recovery after external‐field removal; (iii) high‐speed droplet transport triggered by a charged glass rod. Reproduced with permission [58]. Copyright 2019, Wiley‐VCH. (C) Droplet electrostatic tweezer platform: (i) charge‐redistribution regulation in the droplet‐electrode‐substrate system; (ii) volume‐spanning manipulation, uphill motion, oil media transport, and arbitrary‐trajectory movement enabled by the DEST. Reproduced with permission [204]. Copyright 2022, National Academy of Sciences. (D) Directional droplet driving on surfaces with charge‐density gradients: (i) schematic, and (ii) experimental images. Reproduced with permission [209]. Copyright 2019, Springer Nature.

DEP provides a solution for contact‐free droplet manipulation using non‐uniform electric fields [201]. In this approach, droplets suspended in a denser oil phase are actuated by dielectrophoretic forces generated from patterned electrodes. DEP enables droplet transport, mixing, and sorting while minimizing channel clogging and sample loss, making it particularly attractive for biological assays and chemical synthesis.

Electrostatic forces enable rapid and reversible droplet manipulation via Coulombic interactions [202, 203]. A notable system uses electrostatic charging to actuate droplets on superhydrophobic surfaces [58]. Friction‐electrified droplets undergo reversible wetting‐state transitions (between $∼151.1^{\circ }$ and $∼156.4^{\circ }$) under the electric field (Figure 9B‐i, ii), while asymmetric electrostatic fields can propel droplets at speeds up to ∼20 mm/s (Figure 9B‐iii). Electrostatic manipulation can also be achieved through droplet electrostatic tweezers (DEST), which operate through non‐ontact electrostatic induction [204]. Charge redistribution between voltage‐applied electrodes and grounded substrates generates Coulombic attraction that traps and guides droplets (Figure 9C‐i). DEST supports arbitrary manipulation of nanoliter–milliliter droplets across inclined surfaces and oil media (Figure 9C‐ii), enabling automated microreactions and high‐throughput analysis. Related studies further demonstrate droplet electrotaxis driven by pyroelectric, triboelectric, and piezoelectric charges [205], while electrostatic repulsion has been used for ballistic droplet jumping with programmable trajectories [206]. Beyond droplet transport, electrostatic forces underpin important liquid‐processing techniques such as electrospinning and electrospraying [207, 208]. These techniques bridge liquid manipulation with advanced manufacturing and transform liquids into functional materials.

Surface charge density gradients provide another mechanism for droplet manipulation [209]. Rewritable charge gradients generated by droplet printing on superamphiphobic surfaces enable high‐speed and ultralong‐distance droplet transport without continuous external energy input (Figure 9D‐i, ii). Compared with conventional wettability gradients, this approach achieves faster transport and longer propagation distances across flat, flexible, and vertical surfaces.

Overall, electric‐field‐driven liquid manipulation offers exceptional capabilities such as rapid response, precise reversibility, and versatile programmability. Despite challenges associated with electrode fabrication and surface requirements, ongoing innovations in electrode design and control strategies may address these constraints and expand application frontiers [66, 210, 211].

### 4.3 Optical‐Driven Liquid Manipulation

As a contactless, energy‐focusing, and spatiotemporally programmable stimulus, light provides versatile strategies for liquid manipulation [212, 213, 214]. This section focuses on photoresponsive wettability, photothermal Marangoni transport, optofluidic platforms, and photopyroelectric systems.

Light momentum facilitates optical manipulation through radiation pressure, forming the basis of optical tweezers [215, 216], which can precisely trap and manipulate microscopic particles. While this paradigm has revolutionized fields such as biophysics by allowing single‐cell and biomolecule manipulation [217], radiation pressure forces are easily overwhelmed by the surface tension and viscous forces in larger‐scale liquids. Therefore, incorporating smart‐material mediators is essential for effective energy conversion.

Photoresponsive wettability materials provide a representative strategy for optical liquid control [218]. Azobenzene‐based monolayers enable light‐driven droplet transport through reversible wettability switching induced by trans–cis photoisomerization [62]. When exposed to ultraviolet (UV) and blue light irradiation, the surface free energy changes reversibly (Figure 10A‐i), while asymmetric illumination generates interfacial‐tension gradients that direct droplet motion (Figure 10A‐ii). Similar light‐controlled transport can also be achieved in aqueous systems containing photosensitive surfactants [219, 220], such as the chromocapillary effect [219], where reversible photoisomerization of azobenzene surfactants (AzoTAB) drives droplet motion through light‐induced interfacial‐tension gradients.

**FIGURE 10:**
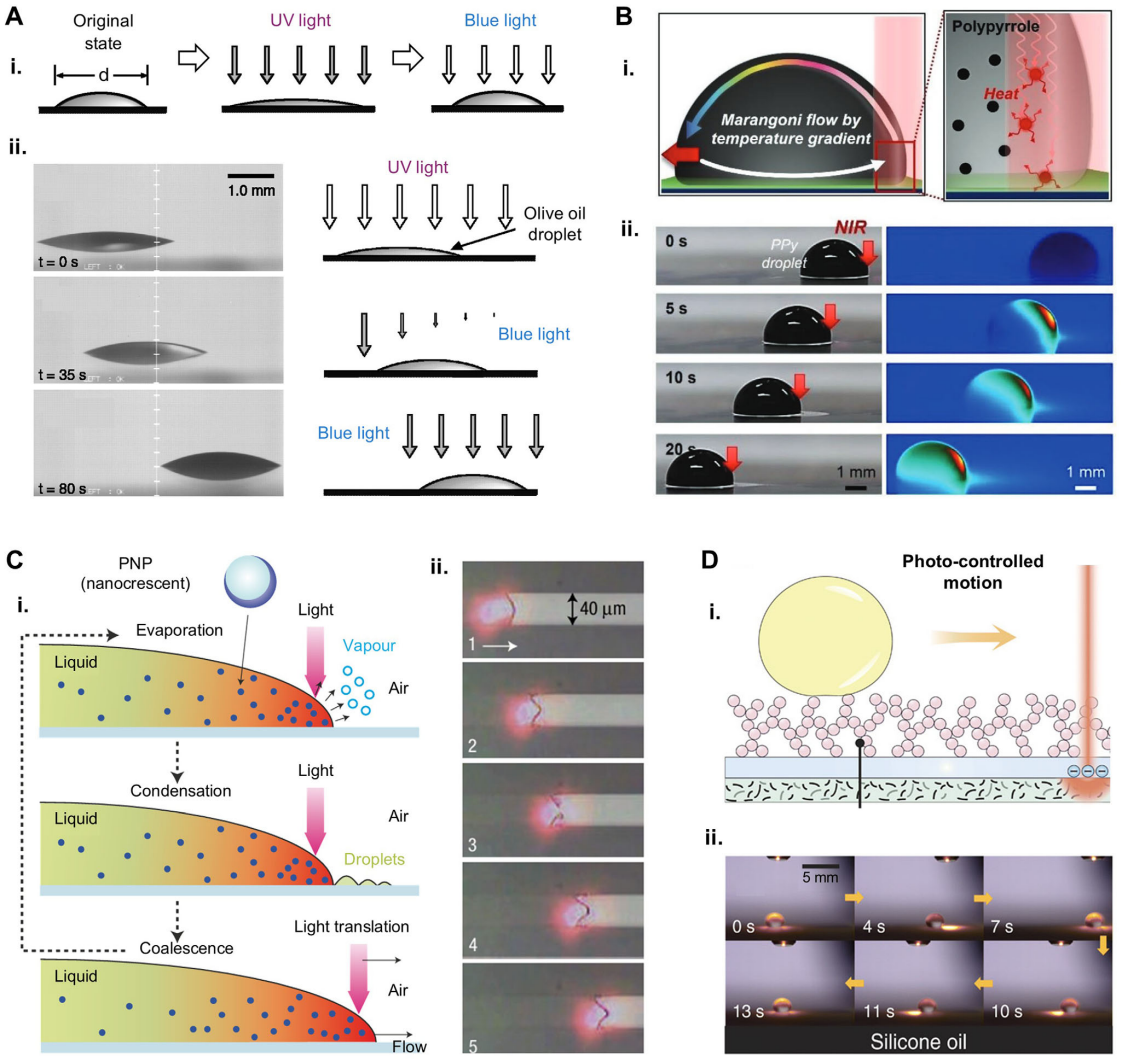
Light‐driven liquid manipulation. (A) Droplet manipulation on photoswitchable wetting surfaces: (i) schematic of UV‐ and blue‐light‐induced wettability changes; (ii) experimental droplet motion driven by a light gradient. Reproduced with permission [62]. Copyright 2000, AAAS. (B) Photothermal driven droplet actuation: (i) schematic of PPy‐laden droplet actuation; (ii) experimental images of NIR‐guided droplet motion and corresponding thermal‐field simulation. Reproduced with permission [227]. Copyright 2022, Wiley‐VCH. (C) Continuous liquid transport in a channel driven by NIR laser: (i) schematic of the rapid localized heating at interfacial photothermal nanoparticles, triggering evaporation, condensation, droplet coalescence, and contact‐line advancement; (ii) continuous laser‐guided liquid transport experiments. Reproduced with permission [228]. Copyright 2006, Springer Nature. (D) Liquid manipulation on a photo‐thermal‐electric platform: (i) schematic of the photo‐thermo‐electric conversion mechanism; (ii) reciprocating motion of silicone oil under light stimulation. Reproduced with permission [230]. Copyright 2020, AAAS.

Photothermal control drives liquid motion through thermocapillary effects [221]. Light absorption can generate thermal surface‐tension gradients that induce microscale liquid transport [222]. This mechanism is particularly effective on lubricant‐infused surfaces [223, 224, 225], where enhanced solid–liquid contact strengthens thermocapillary forces, whereas conventional hydrophobic surfaces show weaker actuation due to limited force transmission [226]. Another strategy generates surface tension gradients directly across droplets using light‐absorbing polypyrrole (PPy) nanoparticles (Figure 10B‐i) [227]. Under localized near infrared (NIR) laser irradiation, PPy‐laden droplets experience internal heating that drives controllable thermocapillary flow and droplet motion (Figure 10B‐ii). Moreover, optofluidic systems utilizing photothermal nanoparticles can drive liquid transport through localized evaporation–condensation dynamics under NIR laser illumination [228]. Rapid localized heating induces droplet coalescence and contact‐line advancement (Figure 10C‐i), and further enables continuous microscale liquid transport (Figure 10C‐ii).

Droplet manipulation can be further enhanced through synergistic coupling of optical, thermal, and electrical fields [190, 228, 229]. A representative photo‐thermal‐electric platform integrates a graphene‐doped heating layer, a pyroelectric lithium niobate substrate, and a superomniphobic surface (Figure 10D‐i) [230]. This light‐driven system generates a wavy dielectrophoretic force field to achieve loss‐free droplet manipulation across wide surface tensions (18.9–98.0 mN/m) and volumes (1 nL–1000 μL), as shown with 5 μL silicone oil (Figure 10D‐ii). Subsequent integration with slippery and superamphiphobic surfaces further expanded multifunctional droplet operations [231, 232, 233].

Light‐driven liquid manipulation provides versatile fluidic platforms by converting optical energy into diverse actuation forces. However, it faces challenges such as limited penetration depth, surface sensitivity, energy inefficiency, and high system cost. Future developments should focus on improving actuation efficiency, programmable high‐throughput liquid processing, and integrated optofluidic platforms through advances in adaptive control systems and specialized materials.

### 4.4 Magnetic‐Driven Liquid Manipulation

Magnetic fields enable non‐contact liquid control with deep penetration and precise spatial control [234, 235]. This section outlines three dominant strategies: magnetic‐particle‐assisted droplet manipulation, ferrofluidic control, and magnetic‐responsive substrates. These methods exploit magnetic forces and torques for programmable liquid handling in soft robotics [236, 237, 238] and microfluidic systems [239].

An early example of magnetic droplet control used magnetic beads embedded within oil‐coated aqueous droplets on hydrophobic surfaces [60]. External magnetic fields enabled controllable droplet motion, coalescence, and splitting, establishing a framework for predictable magnetic droplet operations [240, 241]. Recent studies further developed magnetic robotic platforms using dual steel beads as actuators (Figure 11A‐i, ii) [242]. By tuning magnetic fields, these systems achieve programmable droplet transport, splitting, and rotation, even under confined, inclined, or harsh conditions (Figure 11A‐iii).

**FIGURE 11:**
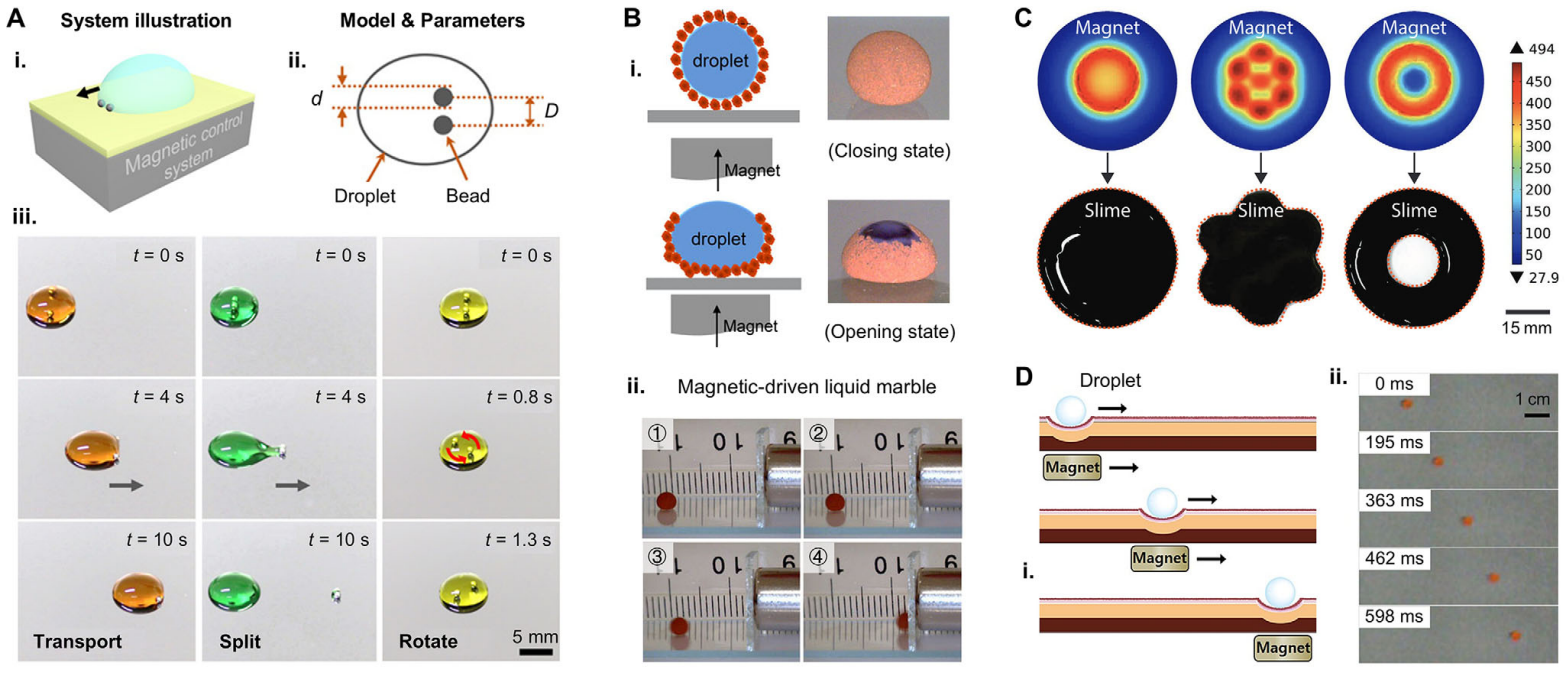
Magnetic‐driven liquid manipulation. (A) Droplet actuation platform using dual steel beads as actuators: (i) schematic; (ii) system model and parameters; (iii)–(v) droplet transport, splitting, and rotation on this platform. Reproduced with permission [242]. Copyright 2020, AAAS. (B) Multifunctional magnetic liquid marbles: (i) magnetically reversible opening and closing of droplets for optical interrogation; (ii) magnetic‐driven motion of a liquid marble. Reproduced with permission [243]. Copyright 2012, Springer link. (C) Programming shapes of ferrofluids using ferromagnets in disk‐, hexagon‐, and ring‐shapes, with the simulation results that show the magnetic field distribution in the plane 2 mm above the magnet. Reproduced with permission [250]. Copyright 2022, Wiley‐VCH. (D) Droplet manipulation on superhydrophobic magnetized surfaces: (i) schematic, and (ii) experiments. Reproduced with permission [253]. Copyright 2013, Wiley‐VCH.

Another strategy employs magnetic liquid marbles, where droplets are encapsulated by hydrophobic magnetic particles [243]. Such liquid marbles can reduce surface adhesion and allow droplet displacement with minimal viscous friction and no leakage [244, 245, 246, 247]. The reversible magnetic encapsulation allows the marble to be opened/closed for optical interrogation of internal chemical processes (Figure 11B‐i). Further, an approaching magnet can trigger the movement of the liquid marble (Figure 11B‐ii). Such systems expand the capabilities of digital microfluidics and show promise for biomedical diagnostics and drug discovery [248].

Ferrofluids, colloidal suspensions of magnetic nanoparticles [249], provide a similar direct magnetic field‐driven manipulation paradigm. They can reshape themselves in response to magnetic fields for navigating terrain obstacles and completing complex tasks (Figure 11C) [250]. Additionally, ferrofluids serve as a medium for “additive‐free” droplet manipulation. Droplets on a ferrofluid‐infused porous surface can be encapsulated by a self‐formed ferrofluid layer and supported by a magnetic‐responsive wetting ridge for droplet transport and programmable processing of micro‐droplet arrays [251]. In addition, ferrofluid‐infused surfaces with reconfigurable topography (i.e., reversibly tunable surface structures) provide integrated functionalities, spanning micrometer‐scale colloidal assembly, millimeter‐scale droplet transport, and centimeter‐scale adhesion control and biofilm removal. These capabilities enable diverse applications in matter manipulation, thermal management, microfluidics, and antifouling systems [252].

Magnetic‐responsive solid surfaces provide an active approach for liquid manipulation by using magnetic‐driven deformation to create mechanical traps or propulsive forces. As illustrated in Figure 11D‐i, ii, superhydrophobic magnetic elastomers can manipulate droplets via moving mechanical traps produced by magnetic‐driven surface deformation [253]. This concept has been extended to droplet mixing systems, which manipulate droplets via programmed membrane deformation and generate internal fluid circulation through back‐and‐forth droplet motion [254]. Inspired by the clearance function of natural cilia [255, 256], magnetic‐responsive micropillar arrays exemplify notable droplet manipulation platforms [61, 257, 258, 259, 260, 261], where the micropillars' dynamic propulsion facilitates precise liquid control. Through tailored surface chemistry and structures, these magnetic‐responsive surfaces find applications in anti‐icing [262], cargo transport [263, 264, 265], soft robotics [266, 267, 268, 269, 270], liquid/solid mixing [271, 272], and antifouling [273].

As a key technology for next‐generation fluidic systems, magnetic actuation provides advantages such as non‐contact operation, excellent biocompatibility, and deep penetration. Despite limitations in spatial resolution and the need for magnetic components, ongoing advances in material design and system integration are broadening their applications in soft robotics, microfluidics, and biomedical diagnostics.

### 4.5 Acoustic‐Driven Liquid Manipulation

Acoustic fields manipulate liquids through controlled sound waves, leveraging intrinsic fluid properties without requiring optical or magnetic responses [274]. This section examines basic mechanisms of acoustic‐based liquid manipulation techniques, including surface acoustic wave (SAW) [275, 276], bulk acoustic wave (BAW) systems [277, 278], acoustic levitation [279], and focal point trapping [280]. These systems are ideal for processing sensitive biological samples and enabling high‐throughput microfluidic applications [185, 281].

Through acoustic‐streaming‐induced hydrodynamic traps, digital acoustofluidics enables programmable droplet manipulation [282]. Initially resting above the ${\text{LiNbO}}_{3}$ substrate, the droplet deforms slightly when trapped, remaining levitated on the oil layer without substrate contact (Figure 12A‐i). Acoustic waves generate counter‐rotating vortices that stabilize the droplet and create hydrodynamic traps upon activation of the interdigital transducer (IDT), which can guide droplet motion for automated biomedical workflows (Figure 12A‐ii, iii).

**FIGURE 12:**
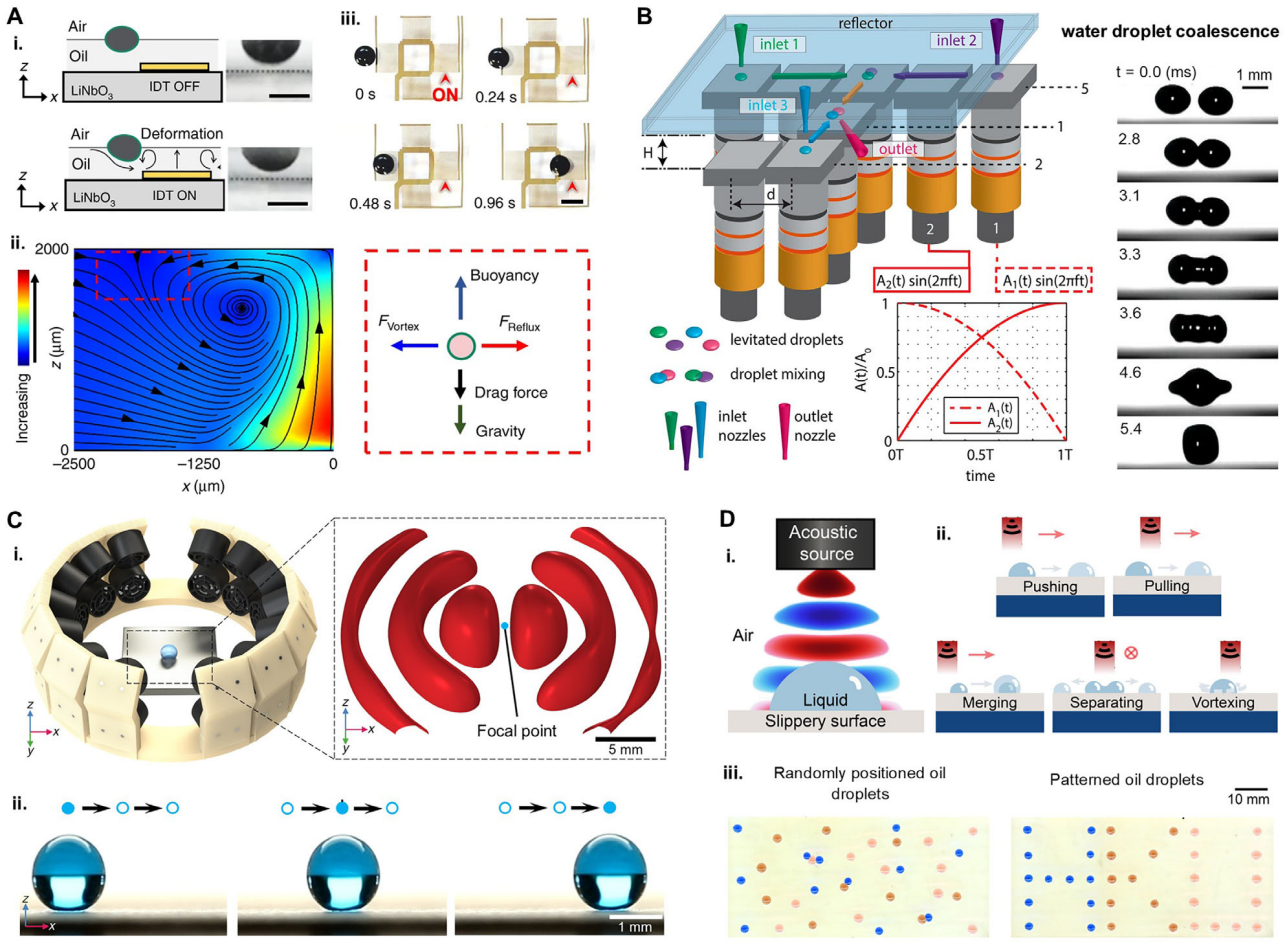
Acoustically driven droplet manipulation. (A) Digital acoustofluidics for droplet handling: (i) comparison of flow fields generated by IDTs in active vs. inactive states; (ii) simulated flow‐field profiles and force diagrams; (iii) experimental droplet motion along hydrodynamic gradients guided by IDTs. Reproduced with permission [282]. Copyright 2018, Springer Nature. (B) Multi‐liquid manipulation on an acoustic‐levitation platform. Reproduced with permission [283]. Copyright 2013, National Academy of Sciences. (C) The ultrasonic tweezer for droplet manipulation: (i) a phased‐array transducer generates a twin‐trap ultrasonic field whose spatiotemporal focal modulation can manipulate droplets on a superhydrophobic surface; (ii) acoustic‐driven droplet movement. Reproduced with permission [63]. Copyright 2023, AAAS. (D) The sound‐controlled fluidic processor: (i) programmable acoustic‐radiation‐force field produced by combining an ultrasonic source with a slippery surface; (ii) various droplet operations via acoustic actuation; (iii) patterned arrangement of randomly positioned oil droplets into the letters “HKU” through acoustic control. Reproduced with permission [287]. Copyright 2025, AAAS.

Acoustic levitation suspends and manipulates droplets in air via precise acoustic radiation pressure. Researchers have created an acoustic levitation platform to generate propagating acoustic traps for near‐constant force during droplet transport [283]. Experiments show the coalescence of two water droplets transported from opposite directions, with schematics indicating potential for complex multi‐droplet processing (Figure 12B). To expand the operable droplet volume range, researchers have developed acoustic tweezers that enable mid‐air levitation of small droplets (less than 20 μL) and in‐plane manipulation of large droplets (up to 500 μL) on superhydrophobic surfaces [284].

Moreover, researchers develop a droplet ultrasonic tweezer (DUT) that can generate a programmable twin‐trap ultrasonic field (Figure 12C‐i) [63], which can manipulate droplets on superhydrophobic surfaces across a wide volume range (0.5–65 μL) (Figure 12C‐ii). Besides, its robust manipulation has expanded to multi‐droplet control [285, 286], applicable in scenarios from ballistic ejection to surface cleaning. Emerging research also notices the importance of surface engineering for enhancing acoustic control. The sound‐controlled fluidic processor integrates an ultrasonic source with an oil‐infused slippery surface to create programmable acoustophoretic force fields (Figure 12D‐i) [287]. This system enables rich fluidic operations (Figure 12D‐ii, iii), for example, pushing/pulling, merging, separating, vortex mixing, and patterning, and the slippery surface provides self‐repairing omniphobicity to minimize liquid adhesion.

Unlike traditional acoustic manipulation relying on superhydrophobic surfaces, the recent acousto‐dewetting technique achieves precise droplet control on superhydrophilic surfaces [288]. It leverages extreme ultrasound confinement within droplets to generate acoustic streaming with a pressure gradient three orders of magnitude stronger than traditional methods, achieving contact line velocities two orders of magnitude higher than previous limits while eliminating viscous film residues.

Acoustic fields provide biocompatibility, material independence, and non‐invasive operation for liquid manipulation. However, challenges related to scalability, energy efficiency, surface interactions, and droplet volume constraints should be addressed. Overcoming these issues will advance acoustic manipulation in intelligent microfluidics, point‐of‐care diagnostics, and advanced manufacturing.

### 4.6 Other Actuation Mechanisms for Liquid Manipulation

Beyond established physical fields, alternative actuation mechanisms provide pathways for liquid control. Pneumatic control is exemplified by a superhydrophobic “aspirator” mesh that use negative air pressure to capture and manipulate tiny droplets [289]. Another method involves programmable super‐repellent surfaces created by embedding microfluidic chambers in a PDMS matrix [290], allowing in situ reconfiguration within 300 ms to guide directional droplet transport and bouncing. Further, a closed‐loop tunable wetting surface combines microfluidic regulation with self‐sensing [291]. This system utilizes an optimized microfluidic chamber network for morphology control, combined with solid–liquid triboelectric sensing for precise droplet tracking, which allows real‐time wettability adjustment during droplet impact for controlled mixing.

Mechanical actuation provides an alternative method to manipulate liquids [292, 293, 294, 295, 296]. Researchers have developed a mechano‐regulated surface with a background mesh and a movable micro‐fiber array featuring different wettability, allowing for reversible alteration of adhesion to liquid droplets [297]. This capability enables lossless handling of micro/nano‐liter droplets in air or immiscible fluids and supports parallel operations. Additionally, adaptive fluid‐infused porous films have been developed for continuous tuning of transparency and wettability [298]. These films utilize a nano‐porous elastic substrate, where mechanical deformation drives liquid flow to control optical and wetting behavior, modulate transparency, and handle low‐surface‐tension droplets, showcasing considerable promise for multifunctional adaptive materials.

Section 4 has examined various active liquid manipulation strategies. These active approaches enable real‐time and programmable liquid control, greatly expanding the functional scope of liquid manipulation beyond what passive architectures can achieve. However, such capabilities typically require continuous energy input and increased system complexity. This trade‐off raises a central question: can liquid manipulation achieve programmability without continuous energy consumption, thereby combining the energy efficiency of passive systems with the adaptability of active control? Addressing this challenge motivates the development of hybrid liquid manipulation strategies, which are discussed in the next section.

## 5 Hybrid Active–Passive Liquid Manipulation: A Synergistic Paradigm

Previous sections have examined passive and active liquid manipulation as two distinct paradigms. Passive systems rely on surface chemistry or structural design to exploit intrinsic interfacial energy for liquid transport, whereas active systems employ external fields to exert forces on liquids. While both strategies have enabled remarkable advances, liquid manipulation faces an intrinsic trade‐off that can be conceptualized as a “liquid manipulation impossible trinity.” An ideal liquid manipulation platform would simultaneously achieve three desirable attributes: (1) zero‐energy operation, enabling autonomous and sustainable transport; (2) programmability and adaptability, allowing dynamic and programmable control; (3) gentle and non‐invasive manipulation, preserving the integrity of sensitive liquids and biological samples. In practice, however, existing approaches typically satisfy only part of these requirements. Passive systems naturally achieve energy‐free operation and often provide gentle liquid handling, but they lack dynamic programmability once fabricated. Active systems offer programmable and real‐time control but require continuous energy input and external fields, which increase system complexity and may introduce disturbances that compromise sensitive liquids or biological environments. This inherent trade‐off defines a central challenge in liquid manipulation.

Recognizing the complementary advantages of passive and active strategies, recent research has increasingly combined passive interfacial architectures with external actuation fields to enhance liquid manipulation performance. In such systems, passive structures guide or rectify droplet motion to improve transport efficiency and precision, while active inputs provide dynamic driving forces and controllability. Several systems illustrate this concept: Leidenfrost droplets on ratchet‐structured hot surfaces exhibit directional self‐propulsion because asymmetric textures rectify vapor flow beneath the droplet [64, 299, 300, 301]; asymmetric interfacial geometries combined with electric actuation in orbital electrowetting platforms enable programmable droplet transport along predefined trajectories [66]; mechanical vibration coupled with anisotropic ratchet surfaces allows rapid and selective droplet transport across wide volume ranges [65]; liquid‐infused soft magnetic carpets switch micropillars between “pin” and “slip” states under magnetic actuation to enable programmable droplet steering [302]. Despite these advances, the driving forces in such systems still arise from continuous external actuation, such that droplet motion remains fundamentally governed by active fields. These approaches therefore represent preliminary explorations toward hybrid liquid manipulation rather than true hybrid architectures.

What, then, defines a true hybrid system? We envision that a true hybrid liquid manipulation system follows a fundamentally different operational principle. In such systems, external energy is used primarily to program the interfacial properties of passive structures, for example by modifying surface morphology or chemical functionality. Once this programming step is completed, liquid motion is governed predominantly by the passive interfacial energy landscape established during programming, with reduced reliance on external energy input. In essence, the energy utilization pathway follows a sequential cascade: external energy → reconfigured interfacial energy (programming) → passive interfacial forces → liquid motion (execution). This programming–execution decoupling enables hybrid systems to combine the energy efficiency of passive mechanisms with the adaptability of active control. By separating the programming stage from the execution stage, hybrid architectures offer a promising pathway toward overcoming the liquid manipulation impossible trinity, enabling autonomous operation, programmable functionality, and gentle, high‐fidelity liquid handling within a single platform.

Corresponding to the branch 3 in Figure 1, hybrid active–passive strategies integrate passive structural design with active field modulation to achieve adaptive and energy‐efficient liquid manipulation. This section surveys representative implementations of such coupled strategies, organized by their underlying coupling concepts rather than technological succession. Although these systems may still remain some distance from the envisioned ideal of liquid manipulation, they nevertheless represent important attempts toward bridging passive and active paradigms.

### 5.1 Coupling Thermal Inputs with Passive Interfacial Architectures for Liquid Control

By integrating structured surfaces, thermally driven liquid manipulation has evolved from basic wettability control to a paradigm capable of guided directionality and temperature‐coupled multi‐directional transport. This section examines how structural design redefines the potential of thermal actuation.

Smart materials are key enablers for adaptive liquid control, and allow seamless integration of active and passive paradigms. An earlier manifestation is the surface modified with thermo‐responsive PNIPAM and networked micro‐textures. Researchers show that surface roughness greatly enhances the thermally responsive wettability of PNIPAM (Figure 13A‐i, ii), which permits reversible switching between superhydrophilicity and superhydrophobicity [67]. This sharp transition stems from the synergistic interplay between active responses and passive surface textures. Following this strategy, a thermal gradient can create a strong wettability gradient to drive droplet motion [303]. In the specific transport zone (30
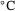
–36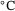), the dramatic changes in CAH generate a net interfacial force capable of propelling water droplets at speeds up to 40 cm/s.

**FIGURE 13:**
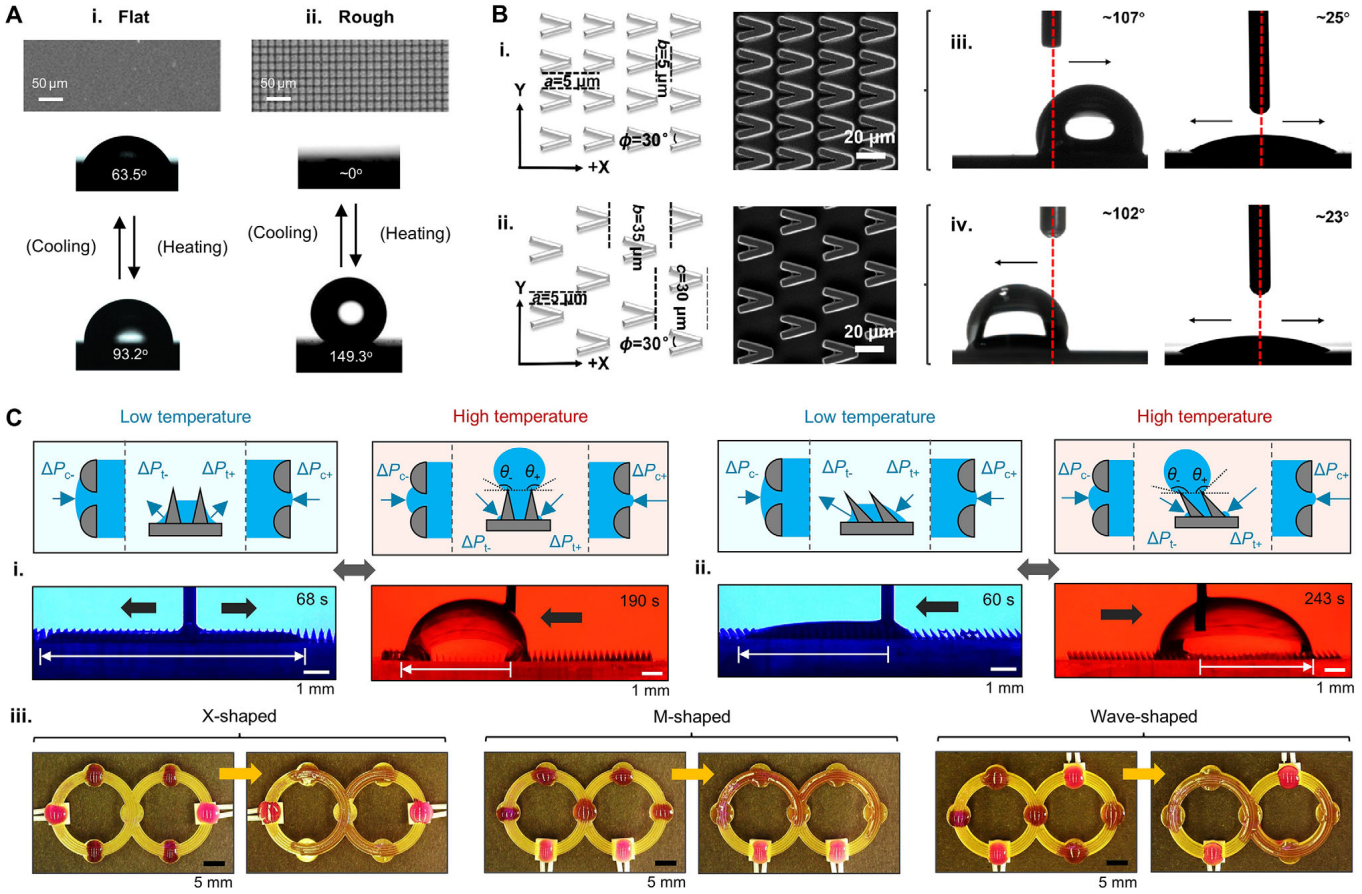
Hybrid active–passive liquid manipulation via thermal fields and structured surfaces. (A) Wettability modulation of PNIPAM‐grafted surfaces under thermal stimuli: (i) flat surfaces exhibit only minor variations in WCAs; (ii) rough surfaces with nanoscale mesh structures show pronounced changes in WCAs [67]. Copyright 2004, Wiley‐VCH. (B) Directional/bidirectional liquid transport on two types of V‐shaped prism microarray surfaces: (i, ii) structural schematics and SEM images; (iii, iv) liquid transport behavior at high versus low temperatures on the two surfaces. Reproduced with permission [70]. Copyright 2019, American Chemical Society. (C) Thermal‐controlled multimodal liquid behavior on dual‐feature PNIPAM‐grafted surfaces: (i) bidirectional‐to‐unidirectional switching on curvature‐only surfaces via temperature change; (ii) directional‐to‐reverse transport on surfaces with both curvature and tilt features under thermal regulation; (iii) programmable liquid patterning on the surface with distributed micro‐heaters. Reproduced with permission [305]. Copyright 2025, American Chemical Society.

On PNIPAM‐grafted surfaces, directional liquid transport can also be achieved through anisotropic structural designs, such as two types of V‐shaped prism microarray surfaces (Figure 13B‐i, ii) [70]. At high temperatures (>LCST), they enable opposite directional control through heterogeneous interfacial resistance, while reverting to bidirectional spreading at low temperatures (<LCST) (Figure 13B‐iii, iv). This strategy has also been extended to pitcher‐plant‐inspired peristome‐structured surfaces to achieve temperature‐controlled directional transport of large droplets [304]. Recently, researchers have introduced dual‐feature (curvature and tilt) 3D heterogeneous surfaces grafted with PNIPAM to program liquid motions [305]. These surfaces facilitate temperature‐responsive switching between bidirectional and directional liquid transport, or even reversal of flow direction (Figure 13C‐i, ii). The system achieves multidirectional liquid control under global thermal fields, while localized heating can redirect flow or enhance uphill spreading on inclines up to 41.8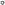. This competitive yet synergistic interplay between thermal fields and structural effects allows programmable liquid patterning (e.g., X‐, M‐, and wave‐shaped) using distributed micro‐heaters (Figure 13C‐iii), supporting cascaded reactions and setting a new approach in open‐surface microfluidic control.

Thermal–passive coupling expands liquid manipulation beyond transport driven solely by temperature gradients or uniform smart‐material responses by introducing spatially encoded structural anisotropy. Integrating thermal inputs with passive architectures enables reversible reconfiguration while preserving directional guidance and energy efficiency. This synergy supports functionalities difficult to achieve through either approach alone, including temperature‐programmable transport direction, switching between directional and bidirectional spreading, and spatially addressable liquid routing under global or localized heating. Passive structures further modulate thermal responses through amplified, biased, or competing wetting effects, enabling adaptive liquid operations in open‐surface microfluidics, thermal management, and programmable chemical processing. More broadly, thermal–passive coupling transforms thermal actuation into a programmable interfacial control strategy.

### 5.2 Coupling Optical Stimuli with Interfacial Passive Elements for Liquid Control

Photoresponsive surface deformation establishes a new paradigm for regulating droplet sliding and directional liquid transport through optically reconfigurable interfacial forces. Coupling optical stimuli with passive architectures enables dynamic control over droplet adhesion, transport direction, and fluidic routing while retaining structural guidance. This integration expands the scope of light‐driven liquid manipulation toward adaptive interfacial fluidic systems.

In a representative study, a plant‐stomata‐inspired surface with programmable wettability arrays is developed for programmable droplet manipulation [306]. The functional substrate, fabricated through coaxial capillary microfluidics, encapsulates graphene oxide (GO) hybrid *N*‐isopropylacrylamide hydrogel within a silica nanoparticle‐dispersed ethoxylated trimethylolpropane triacrylate phase. Upon NIR irradiation, the photothermal response of GO triggers localized shrinkage of hydrogel units, switching the surface wettability from hydrophilic to hydrophobic states (Figure 14A‐i). This reversible transition enables dynamic control over droplet adhesion and sliding trajectories along pre‐designed pathways. By integrating patterned photomasks for spatially selective illumination, the platform shows diverse droplet patterning and transfer‐printing capabilities (Figure 14A‐ii). Beyond simple wettability switching, the combination of photoresponsive deformation and patterned passive architectures enables spatially addressable liquid control, supporting reconfigurable open‐surface microfluidics and programmable liquid handling.

**FIGURE 14:**
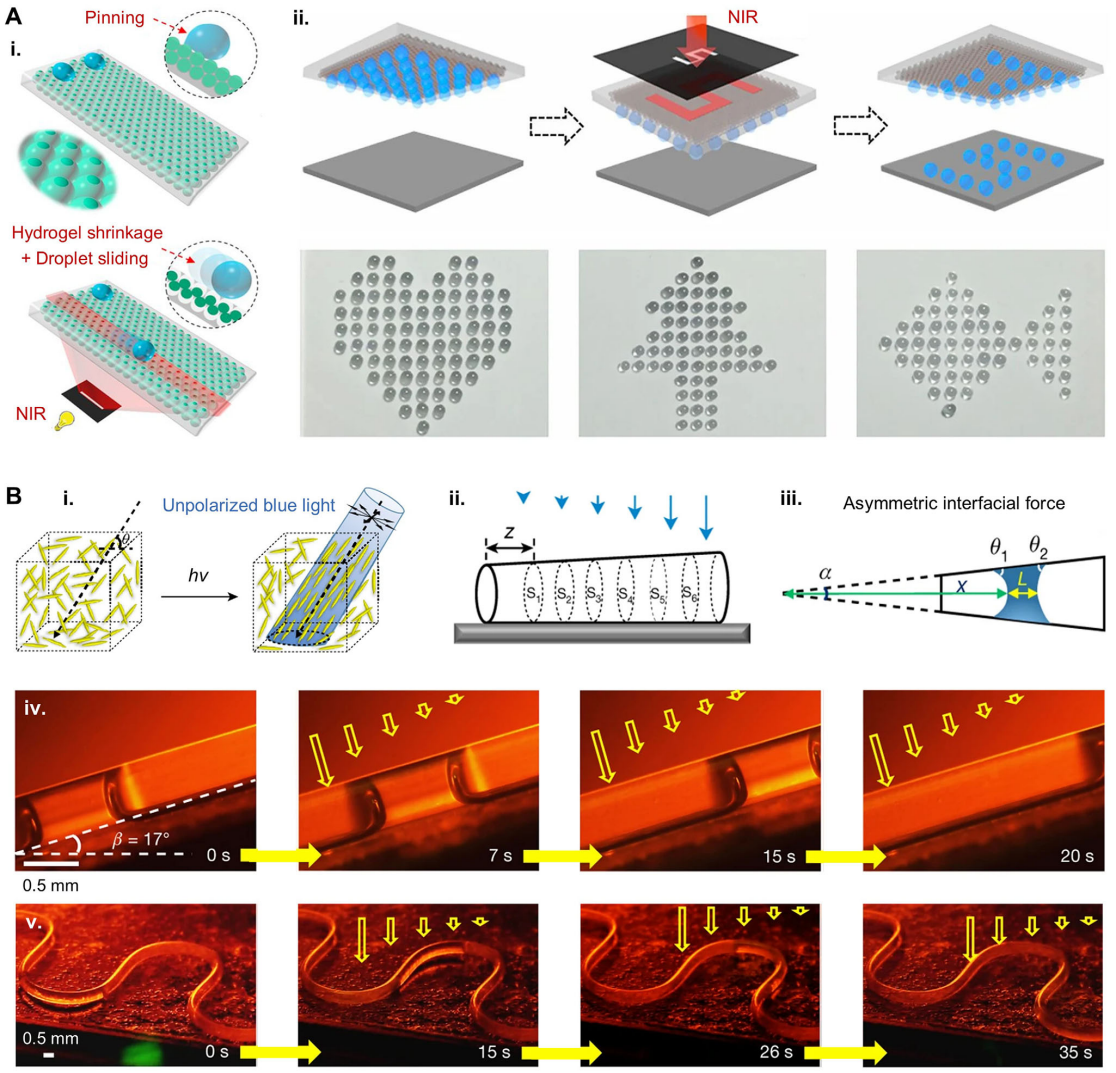
Hybrid liquid manipulation via light‐driven structural morphing. (A) Droplet manipulation on a light‐driven wetting surface: (i) schematic of a pinned droplet on a hydrophilic surface and its sliding after NIR‐induced shrinkage switches the surface to hydrophobic; (ii) multi‐droplet patterning under different optical masks. Reproduced with permission [306]. Copyright 2020, National Academy of Science. (B) Photodeformable tubular microactuators for directional liquid control: (i) schematic of light‐induced liquid‐crystal realignment; (ii) graded light‐induced deformation along the tube; (iii) asymmetric interfacial forces from differential tube deformation that guide liquid directionally; (iv,v) light‐driven uphill motion of silicone oil and its movement along an S‐shaped track. Reproduced with permission [69]. Copyright 2016, Springer Nature.

The introduction of graded photodeformation has expanded the abilities of light‐driven liquid control. A notable development in this direction is the photodeformable tubular microactuator (TMA), fabricated from a mechanically robust linear liquid crystal polymer, which enables direct liquid propulsion through photo‐induced capillary forces [69]. Upon exposure to unpolarized actinic light, the azobenzene mesogens within the TMA reorient along the light propagation direction and induce asymmetric cross‐sectional deformation (Figure 14B‐i, ii). This light‐intensity‐dependent deformation reshapes the microactuator into an asymmetric cone‐like geometry, which in turn creates a programmable capillary gradient that drives liquid slugs toward lower‐intensity regions (Figure 14B‐iii). The TMA can drive silicone oil uphill on a 17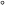 incline at ∼0.1 mm/s and guide liquids along customized paths such as horizontal S‐shaped tracks (Figure 14B‐iv, v), opening avenues for liquid handling in micro‐reactors and lab‐on‐a‐chip systems.

By coupling optical stimuli with passive interfacial architectures, light‐driven liquid manipulation expands beyond conventional photoresponsive wetting or photothermal transport toward structurally encoded and spatially programmable fluidic control. Rather than relying solely on direct optical actuation, passive elements translate optical inputs into reconfigurable interfacial force landscapes through controlled deformation, anisotropy, and capillary asymmetry. This integration enables functionalities difficult to achieve through purely optical or passive strategies alone, including light‐programmable transport pathways, dynamically tunable adhesion states, and remote reconfiguration of capillary driving forces. Passive structures not only mediate energy conversion but also amplify and localize optical responses, enabling efficient liquid operations under spatially selective illumination. Such capabilities are attractive for reconfigurable lab‐on‐a‐chip systems, and programmable chemical synthesis [178, 184]. Optical–passive coupling therefore shifts light‐driven liquid manipulation from localized response behaviors toward programmable interfacial systems with enhanced adaptability and practical utility.

### 5.3 Coupling Interfacial Topography and Magnetic Fields for Liquid Manipulation

A new magnetic manipulation strategy reprograms the surface topography to control interfacial forces, eliminating the constant energy requirements of conventional magnetic‐driven fluidic systems. This hybrid approach uses magnetic reconfiguration of structural features to indirectly regulate liquid behavior. By adjusting the surface geometry, it modulates interfacial forces to guide and pattern liquids. This section examines how magnetically restructured surfaces exploit passive interfacial phenomena to advance adaptive microfluidics, soft robotics, and energy‐efficient liquid handling.

A magnetic fluid/nanoarray composite surface, through field‐induced surface structural transitions, has enabled directional droplet movement [307]. This surface integrates magnetic fluid into a nanostructured array, where alternating magnetic fields drive reversible morphological switching between smooth and rough states for real‐time wettability control and programmable transport. As shown in Figure 15A, water droplets maintain symmetric contact angles ($∼53^{\circ }$) under uniform magnetic fields (12.9 mT), yet instantly develop asymmetric wetting profiles ($\theta_{1}\approx$ 78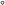, $\theta_{2}\approx$ 38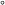) when exposed to gradient fields (21.9–10.2 mT). This directional transition generates controlled wetting gradients to transport droplets with adjustable speeds and trajectories. A related hybrid strategy uses magnetic onoff switching to reconfigure surface topography for tuning droplet adhesion and wetting states [308, 309]. Such interfacial control achieves programmable droplet manipulation and offers a simple yet effective route to adaptive liquid handling.

**FIGURE 15:**
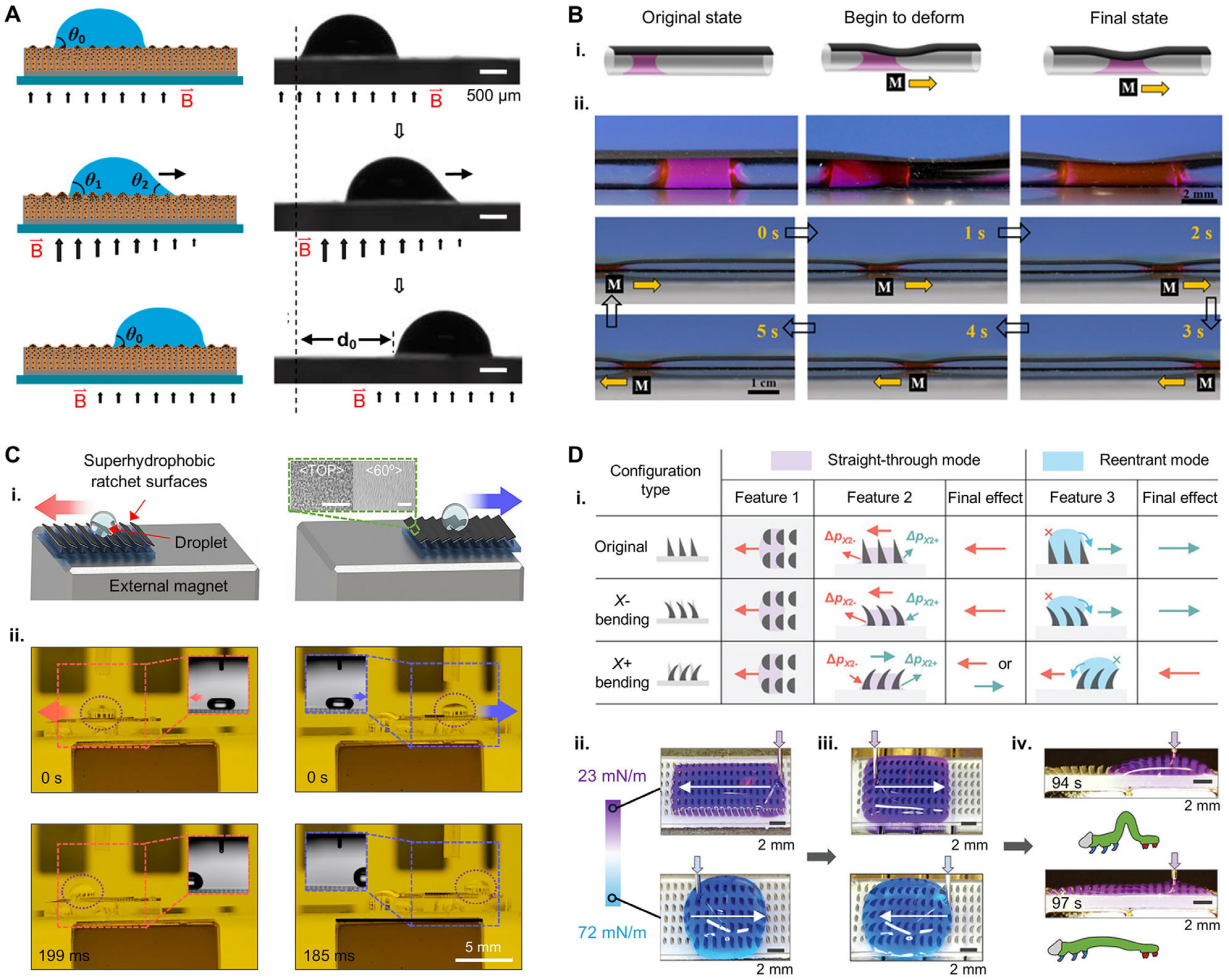
Hybrid liquid manipulation via magnetic‐driven surface morphing. (A) Droplet motion driven by morphological switching of a magnetic fluid/nanoarray composite surface: schematic and experimental images. Reproduced with permission [307]. Copyright 2016, American Chemical Society. (B) Liquid transport driven by asymmetric interfacial forces via magnetic‐driven tubular microactuators: (i) schematic of liquid actuation; (ii) experiments of reciprocating liquid motion. Reproduced with permission [310]. Copyright 2018, AAAS. (C) Controlled directional droplet transport on a magnetic‐driven superhydrophobic ratchet surface: (i) schematic of bidirectional droplet motion enabled by magnetic reconfiguration of the ratchet surface; (ii) experimental demonstration of bidirectional droplet motion. Reproduced with permission [312]. Copyright 2023, American Chemical Society. (D) Multimodal 3D liquid manipulation on magnetic ratchet surfaces: (i) summary of different liquid transport behaviors guided by multi‐feature surface structures under various reconfiguration modes; (ii) selective directional transport of liquids with different surface tensions; (iii) tunable directional transport of liquids with different surface tensions; (iv) inchworm‐like 3D liquid manipulation. Reproduced with permission [71]. Copyright 2024, Wiley‐VCH.

Similar to light‐induced tubular deformation strategies [69], magnetic tubular microactuators provide a parallel method for directional liquid transport by generating asymmetric interfacial forces through magnetic actuation [310]. Constructed from magnetically responsive PDMS, these microtubes undergo programmed shape transformation under moving magnetic fields, forming dynamic conical geometries that establish controlled Laplace pressure gradients to drive liquid movement (Figure 15B‐i). The resulting asymmetric cross‐section creates directional wetting gradients that overcome contact angle hysteresis, enabling precise control over droplet velocity and trajectory (Figure 15B‐ii). This approach achieves propulsion speeds up to 10 cm/s, the fastest reported performance for stimulus‐driven capillary transport in closed tubular systems. This design concept can be extended to other functional materials, such as shape memory slippery tubes [311]. Through a hybrid active–passive strategy, such systems enable autonomous liquid propulsion along preconfigured pathways, while allowing real‐time flow control, including magnetic on/off switching and thermal reprogramming of transport direction.

The synergy between passive structures and active magnetic fields further extends to systems governing droplet impact dynamics, a regime where purely passive surfaces are inherently limited to fixed unidirectional behaviors. Researchers have developed magnetic‐responsive superhydrophobic ratchet surfaces for bidirectional droplet movement control [312]. They consist of silicon strips mounted on elastomeric ridges with superhydrophobic black silicon nanostructures on top and magnetic layers beneath. Under programmed magnetic fields, these strips reconfigure surface topography into bidirectional tilting and thus guide droplet movement directions (Figure 15C‐i). The system operates through tilting‐angle‐dependent Laplace pressure gradients for bidirectional droplet propulsion (Figure 15C‐ii), with horizontal acceleration exhibiting positive correlation to strip inclination angle. This magnetically reconfigurable strategy extends droplet impact control beyond fixed passive ratchets by enabling reversible switching of transport direction and impact behavior for adaptive droplet routing and dynamic liquid redistribution.

Conventional magnetic‐actuated microarray surfaces necessitate continuous field modulation to maintain droplet transport [61, 261, 302]. By contrast, a hybrid strategy combining passive structural guidance with active magnetic steering enables field‐direction‐dependent micropillar tilting for liquid spreading control via unbalanced interfacial forces [68]. This approach eliminates the need for sustained field application, therefore enhancing the intrinsic liquid handling capabilities of passive surfaces while streamlining actuation [313, 314]. Recent advance has extended this paradigm by integrating multi‐feature 3D ratchet surfaces with preprogrammed magnetic fields [71]. In this system, liquid behavior exhibits high sensitivity to variations in both magnetic field parameters (strength/direction) and liquid surface tension (Figure 15D‐i–iii). The reconfigurable microratchets utilize Laplace pressure asymmetry to steer liquids ($10^{0}$–$10^{3}$ μL) across a wide range of surface tensions (23–72 mN/m). This adaptive platform enables multimodal directional transport and even inchworm‐like spreading (Figure 15D‐iv), illustrating how hybrid passive–active architectures are expanding the frontiers of complex 3D liquid manipulation. Meanwhile, it shows how magnetic programming unlocks latent functionalities in passive architectures, allowing liquid behaviors beyond static or continuously actuated systems and expanding the adaptability and complexity of liquid manipulation.

To sum up, these advances shift magnetic‐driven liquid manipulation from direct fluid actuation toward programmable interfacial‐force regulation. Coupling passive architectures with magnetic guidance enables dynamic control over wettability, geometry, and force asymmetry, supporting reversible transport switching, bidirectional control, and multimodal liquid behaviors beyond conventional single‐mode operation. By embedding structural guidance into magnetic actuation, these hybrid systems reduce dependence on continuous field modulation and sustained external forcing, while exploiting reconfigurable interfacial landscapes to expand operational flexibility and simplify liquid manipulation. These properties are advantageous for adaptive capillary routing and closed‐space liquid handling [182], where precise yet remotely tunable interfacial control is required.

### 5.4 Practical Value of Hybrid Liquid Manipulation Systems

While the preceding sections define the conceptual framework of hybrid liquid manipulation and illustrate it through representative examples, an equally important question concerns its practical value: what capabilities do hybrid systems offer that passive or active approaches alone cannot achieve? The key advantage lies in the distinctive operational principle of hybrid systems, namely the decoupling of programming and execution. By using external energy primarily during a programming stage and allowing liquid motion to proceed under stored interfacial energy thereafter, hybrid systems introduce a fundamentally different mode of operation that bridges the strengths of passive and active liquid manipulation.

One major opportunity enabled by this architecture is zero‐power programmability. In conventional passive systems, liquid‐handling behaviors are fixed once surface structures are fabricated, limiting their adaptability to changing tasks. Active systems, by contrast, provide programmable control but typically require continuous energy input and sustained actuation fields. Hybrid systems offer an alternative paradigm in which fluidic operations can be programmed into the static interfacial properties of surfaces. Once programmed, droplets may move, split, merge, or follow predetermined pathways autonomously under the passive interfacial energy landscape. This capability is particularly attractive for energy‐autonomous fluidic systems operating in environments where continuous power supply is impractical or undesirable, such as implantable biomedical devices, remote environmental sensors, or space exploration platforms. In such scenarios, a brief energy input could encode complex, multistep fluidic operations that subsequently execute for extended periods without sustained energy consumption.

A second key advantage is gentle and noninvasive liquid manipulation, enabled by the temporal separation between actuation and liquid motion. In many active liquid manipulation systems, droplet transport relies on continuous electric, magnetic, optical, or thermal actuation. Although effective for fluid control, persistent fields may perturb chemical environments or affect delicate biological samples. Hybrid systems mitigate this limitation by confining external stimuli to a short programming stage. Once programmed, droplet motion proceeds under passive interfacial forces in a field‐free environment, allowing liquids and suspended biological entities to move without sustained exposure to external actuation fields. This feature is particularly valuable in biological and biochemical applications involving fragile cells, proteins, or biomolecular assemblies, where preserving native states is essential for accurate analysis and reliable downstream processing.

Together, these advantages highlight the distinctive value proposition of hybrid liquid manipulation systems. By combining programmable control with energy‐efficient and minimally intrusive operation, hybrid architectures expand the design space of liquid manipulation beyond the capabilities of purely passive or purely active approaches. To further clarify the relationships among these approaches, Table 1 provides a systematic comparison of passive, active, and coupled liquid manipulation paradigms, including their system composition, characteristic scales, energy conversion pathways, response speeds, system complexity, controllability, robustness, functionality, key advantages, and main application domains. This comparison highlights the fundamental distinctions between the three strategies and clarifies the operational contexts in which each strategy is most advantageous.

**TABLE 1:** Comparative analysis of passive, active, and hybrid liquid manipulation systems.

	**Passive systems**	**Active systems**	**Hybrid systems**
Composition	Engineered surfaces	External control systems + Engineered surfaces [optional]	External control systems + Engineered surfaces
Scale	Micrometer to millimeter (surface structures/wetting gradients)	Nanometer to centimeter (field range)	Multi‐scales
Energy conversion pathway	Interfacial energy → Liquid	External energy → Liquid	External energy → Reconfigured interfacial energy → Liquid
Response speed	Millisecond to second (interface‐dependent processes)	Microsecond to millisecond (instantaneous field response)	Millisecond (field‐driven structural deformation)
System complexity	Low (no external components)	High (power supply, signal controllers)	Moderate (simplified external components)
Controllability	Limited flexibility (static/predetermined paths)	High flexibility (dynamic programmability)	Balanced flexibility and stability
Robustness	High (no moving parts)	Moderate (device stability‐dependent)	Moderate to high (enhanced fault tolerance)
Key advantages	Energy‐free operation; structural robustness	Real‐time programmable control	Programmable yet energy‐efficient manipulation; gentle handling
Main applications	Self‐cleaning; water harvesting; condensation management…	Lab‐on‐chip systems; adaptive fluid routing…	Energy‐autonomous fluidics; biomedical assays…

However, it should be noted that hybridization does not eliminate the inherent limitations of individual approaches. Rather, it often integrates their constraints into more sophisticated but still bounded functional designs. In this sense, hybrid strategies should be viewed not as replacements for passive or active methods, but as a valuable expansion of the liquid manipulation engineering toolkit that enables tailored solutions for specific operational contexts.

In the following chapter, we examine how passive, active, and hybrid liquid manipulation strategies perform in different practical scenarios. By evaluating representative applications across multiple domains, we assess which liquid manipulation strategies are most suitable under specific operational requirements and identify opportunities for their complementary use in practical liquid manipulation systems.

## 6 Strategy‐to‐Application Mapping in Liquid Manipulation

This section analyzes typical application domains using a “challenge–strategy–suitability” framework to determine the appropriate selection of passive, active, and hybrid liquid manipulation strategies under different operational requirements rather than representing a fixed technological hierarchy. We focus sustainable energy systems, smart manufacturing, healthcare engineering, as well as other interdisciplinary scenarios where precise liquid manipulation is vital.

### 6.1 Sustainable Energy Applications

#### 6.1.1 Atmospheric Water Harvesting

Amid growing global water scarcity, atmospheric water harvesting has emerged as a promising approach for decentralized water supply, particularly in arid regions with limited infrastructure. Effective systems must capture, transport, and collect condensed droplets under fluctuating humidity conditions while maintaining low energy consumption and scalable deployment.

Passive liquid manipulation strategies are widely adopted in this field because they enable energy‐free water collection through surface structural design and interfacial energy regulation [315, 316, 317]. Inspired by natural fog‐harvesting organisms, for example, spider silk, cactus spines, and desert beetles [9, 318, 319, 320], engineered surfaces incorporate biomimetic wettability patterns and asymmetric structures to promote droplet nucleation, growth, and directional transport [321, 322, 323, 324]. A representative example is a multi‐biomimetic surface integrating beetle‐inspired convex structures, cactus‐like slopes, and pitcher‐plant‐inspired slippery coatings (Figure 16A‐i) [325]. The asymmetric slippery surface greatly improves growth rates and water collection volumes compared to flat surfaces (Figure 16A‐ii). Similarly, biomimetic passive water‐harvesting systems have recently achieved collection efficiencies of up to 60 000 mg/${\mathrm{cm}}^{2}\cdot h$ [326].

**FIGURE 16:**
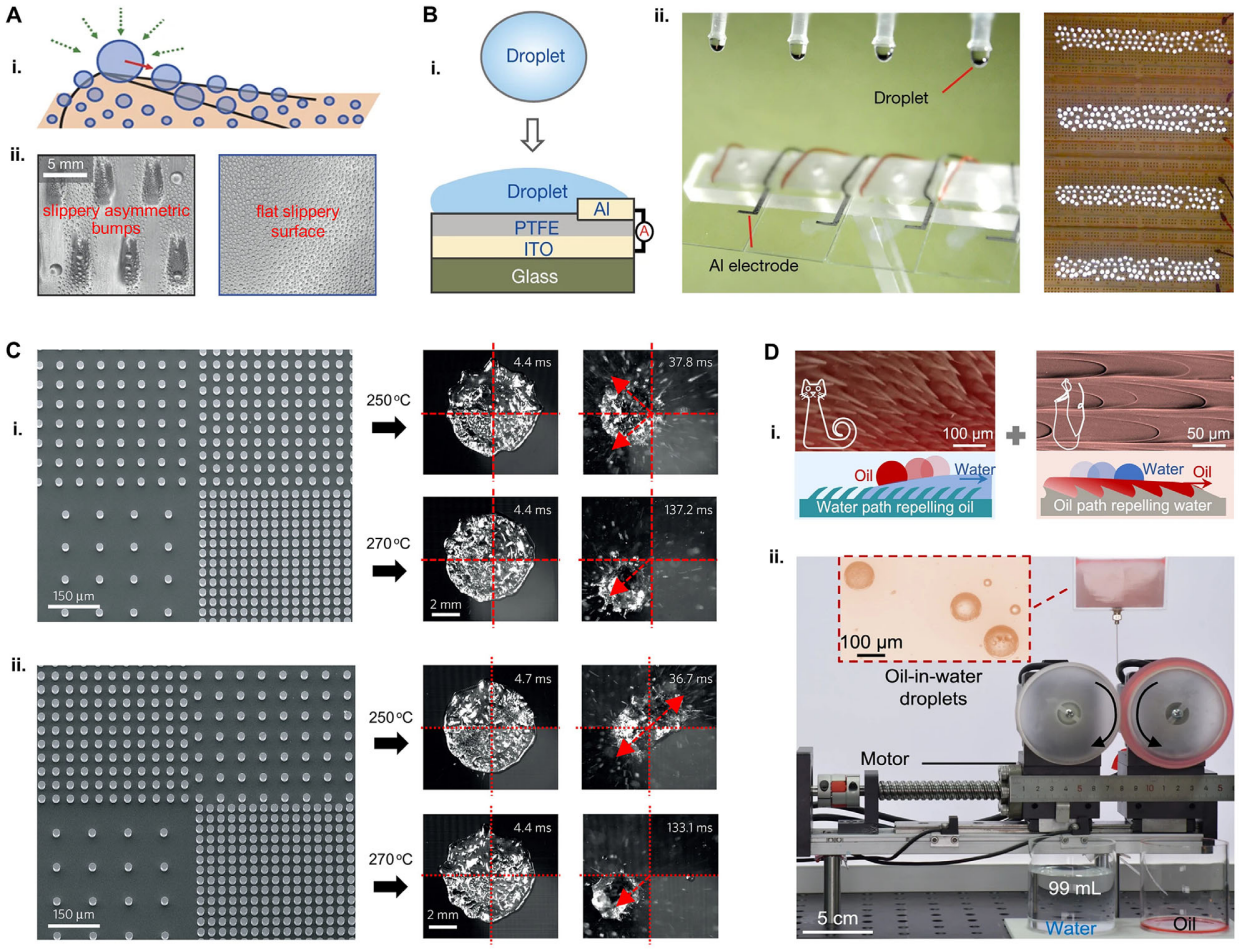
Sustainable energy applications via liquid manipulation. (A) Triply bioinspired water‐harvesting surfaces: (i) design schematic; (ii) experimental comparison of water collection on slippery surfaces with asymmetric bumps versus flat slippery surfaces. Reproduced with permission [325]. Copyright 2016, Springer Nature. (B) Droplet‐based electricity generator for efficient energy harvesting: (i) schematic of the switching‐circuit design; (ii) droplet‐driven power generation experiment lighting 400 commercial LEDs. Reproduced with permission [342]. Copyright 2020, Springer Nature. (C) Droplet transport on structured thermal surfaces: (i) under one design, dropl'ets first move toward the left side with sparse microstructures, then shift toward the sparser bottom‐left corner at higher temperatures; (ii) under a second design, droplets initially move toward the sparser diagonal sides, and later, at elevated temperatures, travel solely along the bottom‐left direction. Reproduced with permission [348]. Copyright 2016, Springer Nature. (D) Passive oil–water separation using dual bio‐inspired structures: (i) schematics of the two bio‐inspired architectures; (ii) experimental setup and actual separation results for oil‐in‐water emulsions. Reproduced with permission [374]. Copyright 2023, Springer Nature.

Active strategies introduce additional control through external actuation such as wind [327], magnetic manipulation [328], or electric stimuli [329]. Although these approaches can enhance droplet transport, their performance often depends on environmental conditions and continuous energy input, and many demonstrations are conducted under high‐humidity laboratory conditions rather than the arid environments targeted for deployment. Moreover, system complexity and cost can limit scalability.

Hybrid concepts have begun to bridge these limitations. For example, a solar‐powered atmospheric water‐harvesting window combines an origami‐structured hydrogel panel with a radiative cooling solar still [330]. Using solar energy for desorption and passive cooling for condensation, the device produces 57–161.5 mL of water per day across humidity levels of 21%–88% during field testing in Death Valley. With a lifespan exceeding 1 year and a competitive cost payback period, this device provides a viable model for scalable, and sustainable decentralized water solutions.

Overall, passive strategies remain the most suitable approach for large‐scale atmospheric water harvesting due to their energy independence and robustness, while active methods offer enhanced control in specific environments. Hybrid systems that combine passive condensation structures with abundant energy sources such as solar radiation [331, 332] and natural wind [333, 334, 335] further improve performance while preserving scalability. Continued advances in materials durability, structural optimization, and standardized evaluation will be essential to translate atmospheric water harvesting technologies from laboratory demonstrations into practical water‐security solutions.

#### 6.1.2 Green Energy Generation

Energy harvesting from ambient water sources has attracted increasing interest as a sustainable pathway for decentralized power generation [336]. Among emerging technologies, droplet‐based electricity generators (DEGs) convert the kinetic energy of falling droplets into electricity through contact electrification at solid–liquid interfaces [337, 338, 339, 340, 341]. When droplets contact functional polymer surfaces, triboelectric charge transfer generates electrical output capable of powering distributed electronic devices.

Active electrical generation mechanisms are central to DEG operation. For example, a switching‐circuit DEG employing a polytetrafluoroethylene (PTFE) layer with an aluminum electrode on an indium tin oxide (ITO) substrate forms a dynamic electrical contact during droplet impact (Figure 16B‐i) [342]. A single 100 μL droplet dropped from 15 cm can generate an open‐circuit voltage of ∼143.5 V and a short‐circuit current of ∼270 μA, corresponding to a peak power density of ∼50.1 W/$m^{2}$ and enabling the illumination of hundreds of commercial LEDs (Figure 16B‐ii).

While active electrical generation mechanisms are central to the DEGs, their synergy with passive interfacial engineering can unlock further capabilities. This coupled strategy can address key challenges in DEGs, such as the formation of a continuous liquid film under high‐frequency droplet impact, which limits charge transfer efficiency and practical utility. As a solution, implementing superhydrophobic surfaces enables rapid droplet shedding and prevents liquid film formation, thus maintains high electrical output even at elevated impact frequencies [343]. Besides, integrating DEGs with triboelectric, pyroelectric, or photovoltaic mechanisms creates synergistic effects that greatly enhance overall performance [344, 345, 346]. These multi‐physics designs improve energy conversion efficiency and broaden related application scenarios.

For energy‐generation applications, hybrid active–passive strategies may provide the most adaptable and efficient solution in the future. By integrating passive interfacial properties with active field‐based actuation, these systems can maintain high energy‐conversion rates across varying conditions while maintaining on‐demand control. Future efforts involve optimizing the synergy between passive interfaces and active stimuli, developing scalable hybrid architectures, and establishing standardized evaluations used in Internet of Things networks, smart agriculture, and autonomous environmental monitoring.

#### 6.1.3 Thermal Management

Efficient thermal management is critical for modern energy systems, where heat dissipation strongly affects performance and reliability [347]. Many thermal technologies rely on droplet evaporation or two‐phase flows to enhance heat transfer, but controlling liquid motion under extreme temperatures remains challenging. In particular, the formation of vapor layers during boiling can cause droplets to become highly mobile yet difficult to direct, reducing heat‐transfer efficiency.

Establishing two concurrent thermal states on a patterned surface can break the wetting symmetry of droplets and induce directional motion [348]. This design creates a Leidenfrost state and a contact‐boiling state side by side, facilitating preferential motion toward the region with a higher heat transfer coefficient. The mechanism of directional droplet motion and confinement is illustrated using a gradient surface morphology. On this surface, structural zones with larger spacings (60 and 100 μm) are situated on the left, while denser zones with smaller spacings (30 and 40 μm) are on the right. As shown in Figure 16C‐i, droplet motion is governed by the interplay between substrate temperature and the local Leidenfrost point. At 250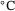, the droplet moves leftward as its right side enters the Leidenfrost state; at the higher temperature of 270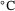, the droplet is directed toward the zone with a spacing of 100 μm, which maintains a contact‐boiling state. Consequently, this approach enables precise droplet localization, whether through diagonal vectoring, elliptical deformation, or zonal confinement (Figure 16C‐ii). This sophisticated coupling of passive topographic patterning with active thermal control allows for the precise manipulation of droplet dynamics under extreme conditions, and holds promise for applications that demand high thermal efficiency and fidelity.

In a complementary manner, the two‐phase thermal regulator utilizes passive capillary structures and Tesla valves (fluidic diode that imparts flow resistance heterogeneity [349, 350]) to rectify vapor–liquid flow at the system scale [16], achieving a similar synergy between surface design and phase‐change thermodynamics. The former controls discrete droplets through asymmetric boiling states; the latter stabilizes continuous two‐phase flow through directional fluidic resistance, together showing the versatility of structure‐guided thermal management strategies.

The integration of passive structural design and active thermal control emerges as a powerful strategy for advanced thermal management. This hybrid approach achieves the efficient and robust performance required to meet escalating thermal challenges, providing a scalable pathway for modern energy systems.

#### 6.1.4 Oil/Water Separation

Oil/water separation is essential for addressing global challenges in industrial wastewater treatment and environmental remediation [351, 352, 353, 354]. Efficient separation requires precise control of liquid transport and wettability to selectively guide oil and water phases [355, 356, 357, 358]. Achieving robust and scalable separation across diverse oil/water mixtures remains a key challenge.

Passive wettability‐engineered materials provide a widely adopted solution for oil/water separation. Oil‐removing materials exhibit superhydrophobic and superoleophilic properties, allowing oil to pass through while repelling water, as demonstrated in sprayed stainless‐steel meshes and other micro/nano‐structured surfaces modified with low‐surface‐energy coatings [359, 360, 361, 362]. However, these materials are prone to oil fouling. Alternatively, water‐removing materials with superhydrophilicity and underwater superoleophobicity can resist fouling and enable gravity‐driven separation of lighter oils [363]. Despite these advances, both approaches face limitations in separating complex mixtures, particularly heavy oils and stable emulsions.

Smart materials with switchable wettability between oil‐ and water‐removing modes have been developed using Janus materials with asymmetric chemical compositions or structures [364, 365, 366], as well as hybrid systems combining passive designs with external actuation [367, 368, 369, 370, 371, 372, 373] (heat, light, electric fields, magnetic fields, or pH) to dynamically alter surface wetting states. While these materials offer great potential, they also introduce increased manufacturing complexity, energy consumption, and operational challenges.

For large‐scale environmental applications, robust passive architectures remain the most practical strategy. For example, a dual‐bionic superwetting gear overflow system integrates surface superwettability with complementary topological structures to achieve directional liquid steering and efficient separation of oil‐in‐water emulsions (Figure 16D‐i, ii) [374]. Based on surface energy matching, oil and water preferentially spread on different gear surfaces to form separate liquid films that are rapidly transported and collected, enabling high separation flux and stable performance across oils with varying densities and surface tensions.

Overall, passive wettability‐engineered systems provide the robustness and scalability required for practical oil/water separation, while active and hybrid strategies offer opportunities for adaptive separation of complex mixtures and emulsions. Future developments should focus on improving fouling resistance, structural durability, and operational simplicity to enable cost‐effective industrial deployment.

Section 6.1 illustrates how liquid manipulation contributes to sustainable energy applications. Passive interfaces play a foundational role by offering robustness, scalability, and versatility across diverse environments. Although continuous active inputs are not always necessary, their integration with passive designs can enhance adaptability in specific scenarios. Future progress will require coordinated advances in energy utilization, passive interface architecture, intelligent materials, and system‐level integration, enabling more efficient and context‐responsive energy technologies.

### 6.2 Smart Manufacturing

#### 6.2.1 Advanced Interfacial Materials

Beyond the abovementioned sustainable and energy applications, advanced interfacial materials have also extended to smart manufacturing. Through multi‐scale structural design and surface chemistry, these systems (e.g., superwetting surfaces [375] and liquid‐infused interfaces [376]) acquire essential industrial capabilities including self‐cleaning, anti‐icing, drag reduction, corrosion resistance, and biointerfacing. These enhanced functionalities establish their vital role in next‐generation manufacturing technologies.

Passive liquid manipulation strategies dominate advanced interfacial materials because structural wettability control can regulate liquid behavior without external energy input. By tailoring surface micro/nano‐structures and chemistry, these interfaces enable diverse manufacturing functions, including self‐cleaning, antifouling, and liquid repellency. For example, lotus‐leaf‐inspired superhydrophobic surfaces combine hierarchical micro/nano‐structures with low surface energy to enable droplet rolling and contaminant removal [90, 377, 378, 379]. Alternatively, superhydrophilic coatings allow water to spread into thin films that wash away contaminants [380]. Biomimetic designs inspired by gecko setae and marine organisms extend these concepts to antifouling and self‐cleaning interfaces [381, 382, 383, 384, 385]. In addition, superoleophobic surfaces constructed from hierarchical textures and fluorinated coatings enable resistance to oils and complex liquids [386].

Similar passive strategies are applied in anti‐icing technologies. Superhydrophobic surfaces reduce ice adhesion by trapping air pockets and minimizing solid–liquid contact, delaying droplet freezing and preventing ice accumulation [387, 388, 389, 390]. For instance, a superhydrophobic coating, made from a PDMS microstructure integrated with zinc oxide nanohairs [391], shows exceptional water and ice repellency at low temperatures ($-20^{\circ }C$) and high humidity (90%) for over 3 months (Figure 17A‐i, ii). SLIPSs offer an alternative strategy by maintaining a lubricating liquid layer that significantly reduces ice adhesion [392]. In more demanding conditions, hybrid strategies integrating passive wettability with active actuation have been explored, such as electrothermal–pneumatic systems that enable efficient deicing under extreme cold [393].

**FIGURE 17:**
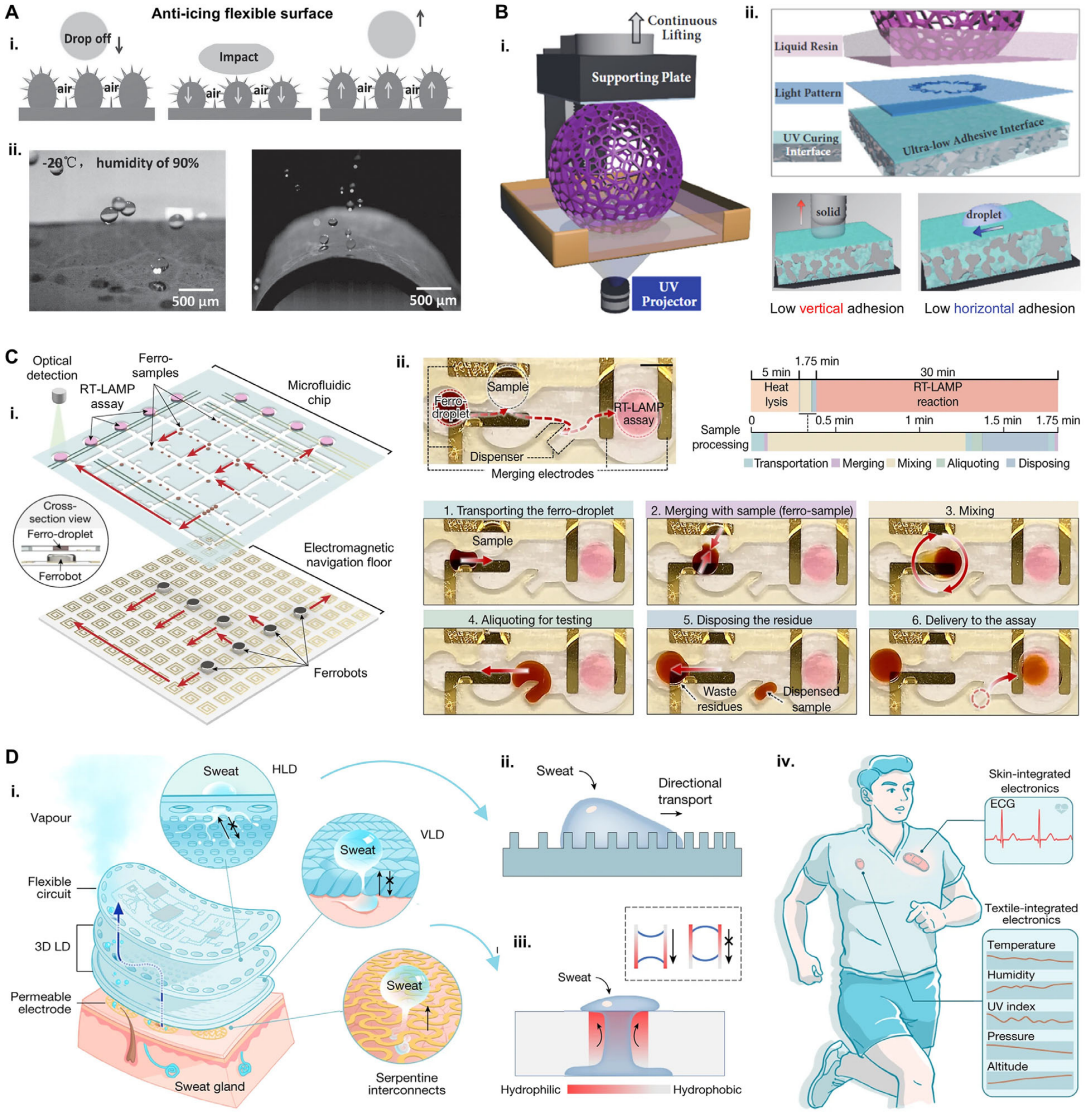
Smart manufacturing enabled by interfacial liquid manipulation. (A) Flexible superhydrophobic surfaces for anti‐icing: (i) schematic of hydrophobic hierarchical surface structures; (ii) anti‐icing experiment under low‐temperature, high‐humidity conditions. Reproduced with permission [391]. Copyright 2016, Wiley‐VCH. (B) Bioinspired slippery surfaces for continuous 3D printing: (i) schematic of continuous printing; (ii) reduced adhesion in vertical and horizontal directions, coupled with rapid resin refilling enabled by slippery surfaces, which enhances printing speed while maintaining performance. Reproduced with permission [416]. Copyright 2018, AAAS. (C) Millimeter‐scale ferrobots manipulating magnetized sample droplets to execute high‐fidelity, flexible nucleic‐acid amplification workflows: (i) schematic of the system setup and operation; (ii) experimental demonstration of complex detection. Reproduced with permission [427]. Copyright 2022, Springer Nature. (D) 3D liquid diode enabled user‐friendly wearable devices that pump sweat unidirectionally away from skin while maintaining dry skin contact and stable biosignal acquisition: (i) device schematic; (ii, iii) multi‐level directional liquid transport structures; (iv) illustration of the wearable application. Reproduced with permission [20]. Copyright 2024, Springer Nature.

Passive interfacial materials are also widely used in drag reduction and corrosion protection. Superhydrophobic surfaces reduce fluid friction by maintaining gas layers at the solid–liquid interface [394, 395, 396], while studies in coated pipelines demonstrate substantial drag reduction, particularly in turbulent flow regimes [397, 398]. To improve stability under high pressure, designs such as electrolysis‐assisted hydrophobic microstructures can maintain gas layers even at elevated pressures [399]. For corrosion protection, superhydrophobic coatings trap air pockets that isolate metal surfaces from water [400, 401]. While SLIPS‐based coatings provide improved resistance through lubricant‐infused barriers [402, 403]. Further refinements in lubricant retention, mechanical durability, and manufacturing scalability are needed to deploy these SLIPS‐based anticorrosion technologies in marine environments [404, 405].

In biomedical manufacturing, interfacial wettability also governs cell–material interactions and device functionality [406]. Surface wettability is crucial in mediating cell–material interactions [407, 408], with superwetting surfaces engineered to display cytophobic (superhydrophobic) or cytophilic (superhydrophilic) properties for selective cell adhesion and manipulation. SLIPSs can also enhance the functionality of medical devices. For instance, SLIPS‐coated endoscopes maintain optical clarity and antifouling capability even after repeated exposure to blood/mucus [409]. These superior interfacial properties extend device lifespan and contribute to safer medical implants and diagnostic systems.

While various interfacial materials have emerged, passive systems based on structural and chemical design maintain the most practically viable. In contrast, hybrid active–passive strategies emphasized in this review have been underexplored. Their promising applications lie in specialized biomedical domains, involving responsive systems (to magnetic fields, pH, and temperature) combined with effective structural design for precise control of microscale biological environments. Despite their complexity, they justify the added costs through enhanced functionality/practicality in high‐value applications.

#### 6.2.2 Interfacial Control in Additive Manufacturing

Interfacial control is a crucial aspect governing the precision and functionality of additive manufacturing by regulating liquid–solid interfacial behaviors [410]. It ensures high‐resolution fabrication while maintaining structural integrity across diverse manufacturing processes. For instance, engineered superhydrophobic nozzles in material jetting [411] minimize adhesion to achieve precise droplet deposition [412], while binder jetting [413] utilizes controlled wetting kinetics and homogeneous powder distribution to enhance binding quality and mechanical performance [414]. Notably, bioinspired slippery surfaces have enabled continuous vat photopolymerization [415]. These liquid overlayer interfaces prevent adhesion during curing, and promote fast resin refilling and continuous printing platform lifting (Figure 17B‐i, ii). This allows for the fabrication of complex 3D architectures within minutes instead of hours [416], highlighting how advanced interfacial engineering can overcome basic limitations in manufacturing processes.

Collectively, these interfacial strategies bridge digital design with physical realization across advanced manufacturing technologies. Continued refinement in interface manipulation will not only improve existing systems but open pathways toward intelligent and efficient production paradigms [417, 418].

#### 6.2.3 Emerging Robotics via Interfacial Liquid Control

At small scales, interfacial liquid control is essential for robotic functionality, as interfacial forces start to prevail over inertial effects [23, 238]. Precise control of surface wettability and interfacial tension therefore provides an effective strategy for enabling locomotion, manipulation, and adaptive behaviors in miniature robotic systems.

Passive surface engineering provides a route to regulate robot‐fluid interactions. Wettability modification can reduce liquid adhesion and hydrodynamic resistance, improving mobility in aquatic environments. For example, hydrophobic artificial bacterial flagella exhibit higher step‐out frequencies and forward velocities than hydrophilic designs [419]. Wettability‐controlled nanoflask motors also demonstrate reversible motion driven by chemical gradients, where hydrophilic and hydrophobic surfaces promote opposite propulsion directions [420]. Such strategies highlight how passive interfacial design can guide propulsion mechanisms at the microscale.

Active strategies further expand robotic capabilities by dynamically regulating interfacial properties through external stimuli. Photothermally responsive PNIPAM hydrogels, for instance, undergo large volumetric and wettability changes at $∼32^{\circ }C$, enabling oscillatory deformation and propulsion in soft robotic systems [421]. External stimuli can also modify local interfacial tension to generate Marangoni flows that propel floating robots [422, 423]. Another active approach employs programmable fluids such as ferrofluids, whose morphology and motion can be controlled by magnetic fields to enable splitting, merging, and navigation in confined spaces [250, 424, 425, 426]. In practical systems, millimeter‐scale ferrobotic swarms can manipulate magnetized droplets to perform automated biochemical workflows, including nucleic acid amplification assays, on compact platforms (Figure 17C‐i, ii) [427]. While these active methods provide high programmability and functional versatility, they typically rely on continuous external actuation.

As mentioned above, future swimming robots will benefit from hybrid passive–active strategies that combine passive surface engineering with active external control. The study on acoustic hydrogel microrobots also exemplifies this approach [22], with its dual‐opening bubble‐trapping cavity ensuring stable propulsion in biofluids and its hierarchical surface design (hydrophobic inner layer for retention, hydrophilic outer against aggregation) guaranteeing operational persistence. This synergy of passive surface engineering and active field‐driven control demonstrates a viable blueprint for biomedical microrobots.

#### 6.2.4 Functional Textiles and Wearable Electronics

Functional textiles [428] and wearable electronics [429] increasingly rely on interfacial liquid manipulation to regulate moisture transport and maintain stable device–skin interfaces. These systems must simultaneously manage sweat, thermal comfort, and signal stability, posing challenges for conventional textile materials.

Janus fabrics have been developed for moisture and thermal management to enhance human comfort and performance [430, 431, 432]. They can transport sweat from the hydrophobic side to the superhydrophilic side to keep the inner surface dry and prevent clingy sensations. A notable case along the focus is a hybrid liquid management approach that integrates external stimuli (i.e., body heat) into the existing passive Janus design to enhance its intelligent properties [433]. One study introduces a thermo‐responsive smart fabric utilizing amino‐silica@PDVB/PNIPAM Janus particles. The fabric features hierarchical micro/nano‐structures, wherein the PNIPAM component undergoes reversible hydrophilic‐hydrophobic switching at $∼32^{\circ }C$, autonomously pumping sweat from the skin (hydrophobic) to the exterior (hydrophilic). This mechanism facilitates rapid evaporation, achieves a perspiration rate five times higher than commercial cotton, and maintains performance over 20 thermal and adhesion cycles.

Interfacial liquid control is also crucial for wearable electronics, where excessive sweat accumulation can degrade device performance and biosignal accuracy. A 3D liquid‐diode architecture based on heterogeneous wettability interfaces enables directional sweat transport away from the skin, achieving pumping rates up to $11.6\;\text{mL}\;{\text{cm}}^{-2}\;\min^{-1}$ while maintaining stable biosignal acquisition (Figure 17D‐i–iii) [20]. This technology has been demonstrated in both skin‐integrated and textile‐integrated electronics (Figure 17D‐iv), which paves the way for scalable, user‐friendly wearable devices.

Future development should focus on harnessing accessible energy inputs such as body temperature and osmotic gradients, while designing cost‐effective interfacial structures. The selection of readily available energy sources and scalable fabrication routes is essential to translate these intelligent liquid‐handling technologies into practical and large‐scale applications.

Section 6.2 shows how passive, active, and hybrid liquid manipulation strategies are empowering a transformative phase in smart manufacturing. Moving forward, a focus on hybrid strategies, which merge programmable responsiveness with durable interfacial properties, will bring exciting opportunities in robust interfacial materials, advanced biomanufacturing, and next‐generation wearable devices.

### 6.3 Healthcare Engineering

#### 6.3.1 Hemostasis and Wound Healing

Effective management of bleeding and wound healing remains a major challenge in healthcare, requiring coordinated control of hemostasis, infection prevention, and tissue regeneration [434, 435, 436]. Traditional wound dressings address only isolated aspects of this process. However, recent advances in interfacial liquid manipulation enable multifunctional wound‐care systems that regulate blood absorption, fluid transport, and therapeutic delivery through engineered surface architectures.

Passive interfacial engineering provides a promising strategy for such applications. In particular, Janus structures with asymmetric wettability can simultaneously regulate liquid transport and biological interactions [437, 438]. For example, a polycaprolactone–chitosan Janus membrane developed for intraoral wound care integrates a hydrophobic outer layer that suppresses bacterial adhesion with a hydrophilic chitosan sponge that rapidly absorbs blood and promotes coagulation (Figure 18A) [439]. This design enhances hemostatic performance and accelerates gingival healing in tooth‐extraction models. Similarly, a hydrogel‐based Janus bandage with gradient wetting channels enables unidirectional drug delivery to the wound bed with over 90% efficiency while maintaining flexibility under skin deformation (Figure 18B) [440]. Such systems promote epithelialization, collagen deposition, and rapid wound closure.

**FIGURE 18:**
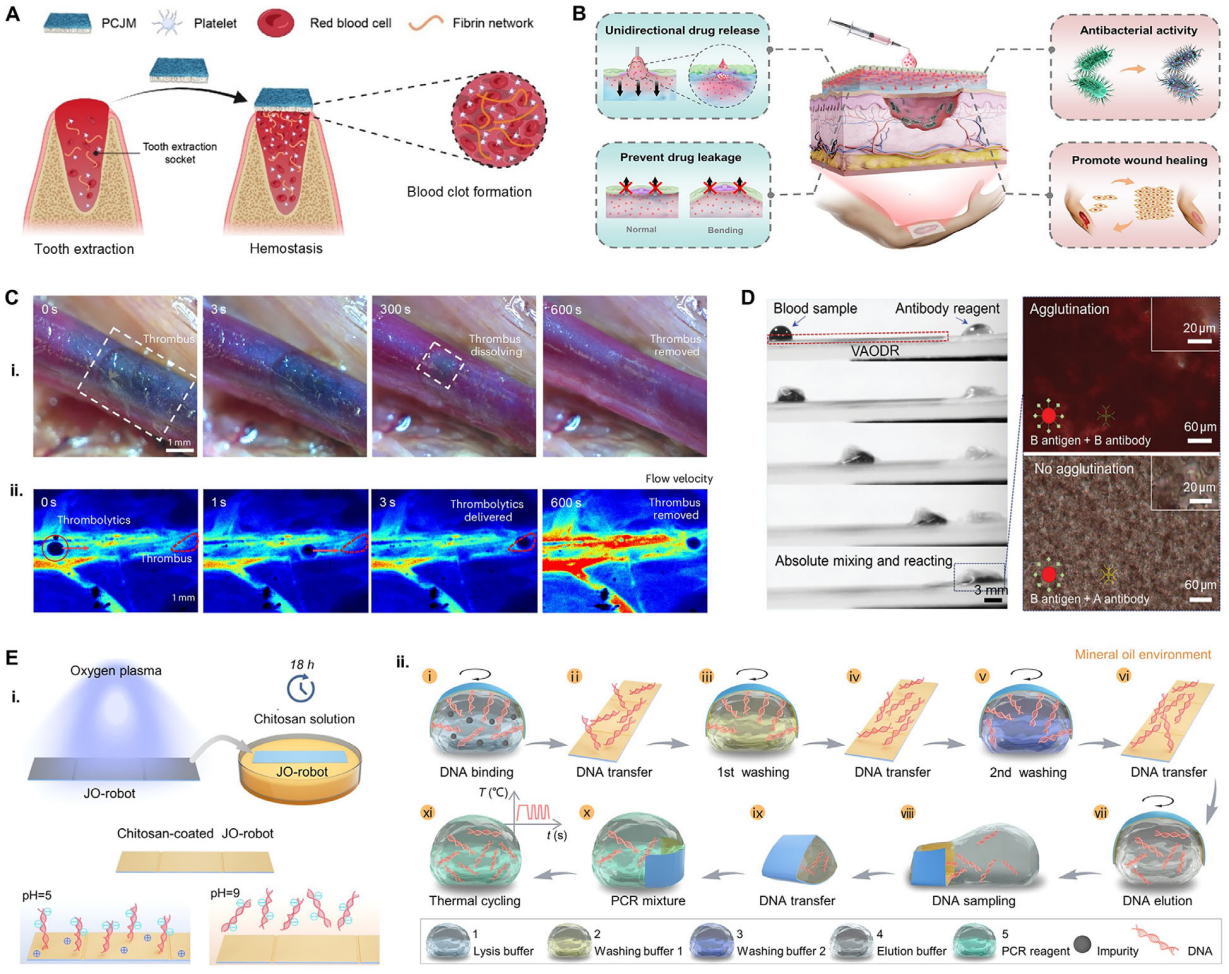
Healthcare engineering applications based on interfacial liquid control. (A) The polycaprolactone–chitosan Janus membrane for intraoral wound care. Reproduced with permission [439]. Copyright 2024, Elsevier. (B) The Janus bandage for chronic‐wound healing. Reproduced with permission [440]. Copyright 2024, American Chemical Society. (C) Thrombus dissolution via acousto‐dewetting: (i) experimental image; (ii) monitored flow‐field image. Reproduced with permission [288]. Copyright 2025, Springer Nature. (D) The vibration‐actuated omni‐droplet rectifier for ABO blood‐group testing. Reproduced with permission [65]. Copyright 2022, Wiley‐VCH. (E) The magnetically actuated Janus origami robot for multi‐step nucleic‐acid extraction and purification in biomedical applications: schematics of (i) the robot and (ii) the application process. Reproduced with permission [241]. Copyright 2023, Springer Nature.

These examples highlight the potential of passive interfacial engineering in developing multifunctional wound dressings. Future integration of localized active stimuli (temperature, pH, or biochemical cues), with bio‐interfacial materials will enable adaptive wound‐care systems. This hybrid active–passive approach promises to transform wound management into a more precise and personalized therapeutic process.

#### 6.3.2 Drug Delivery

Effective drug delivery requires precise control over the spatial distribution and release of therapeutic agents [441, 442, 443]. However, complex biological environments often lead to premature drug release, insufficient targeting accuracy, or inadequate local drug concentrations. Interfacial liquid manipulation provides important tools to address these challenges by regulating droplet transport, adhesion, and release through engineered surface chemistry, microstructures, and wettability. In addition, external stimuli such as acoustic, optical, magnetic, and electrical fields have been introduced to improve spatiotemporal precision and adaptability in biological environments [444, 445, 446].

Active interfacial control strategies are particularly useful for targeted delivery in complex tissues. For example, acousto‐dewetting combines acoustic actuation with interfacial liquid control to manipulate droplets on surfaces of arbitrary wettability, including highly hydrophilic biological tissues [288]. Acoustic streaming generates strong pressure gradients that enable precise 3D droplet transport without surface modification. In an in vivo demonstration, a thrombolytic droplet is guided to a thrombus site in a rat femoral vein, where controlled droplet motion along the vascular wall enables localized drug action and restores blood flow within 600 s (Figure 18C‐i, ii).

Future systems may integrate such active control with responsive biomaterials to realize hybrid active–passive delivery platforms [447]. They use external actuation for spatial targeting and triggered release, while engineered interfaces regulate pharmacokinetics and biocompatibility. Such interfacial liquid control strategies will pave the way toward personalized adaptive drug delivery systems.

#### 6.3.3 Diagnostic Sample Processing

Miniaturized liquid handling is central to modern diagnostic technologies, where precise manipulation of microliter‐scale samples determines detection sensitivity, accuracy, and throughput [448, 449]. Diagnostic assays typically involve multiple steps, including mixing, transport, separation, purification, and reaction, which must be executed reliably within compact platforms. Achieving programmable multi‐step operations while maintaining robustness and biocompatibility remains a key challenge.

Passive interfacial strategies can support simple liquid operations through engineered wettability and surface structures that guide droplets without external energy input. However, their static nature limits their ability to perform complex or programmable biochemical workflows. Active strategies therefore play a crucial role in diagnostic sample processing. For instance, a vibration‐actuated omni‐droplet rectifier combines a slippery ratchet surface with programmable vibration to achieve directional transport and rapid mixing, accelerating ABO blood‐group testing through enhanced blood–antibody mixing and faster agglutination detection (Figure 18D) [65]. More complex diagnostic workflows can be realized by combining programmable actuation with functional interfacial materials. A magnetic‐actuated Janus origami robot enables droplet transport, splitting, dispensing, and stirring through magnetic control [241]. The robot integrates chitosan‐modified regions for pH‐dependent nucleic‐acid adsorption with superhydrophobic regions for droplet manipulation (Figure 18E‐i). Under magnetic actuation, this system performs multistep nucleic‐acid extraction and purification, followed by sample dispensing for downstream PCR analysis (Figure 18E‐ii).

In the future, hybrid liquid‐control strategies that combine the robustness of passive interfacial design with the flexibility of active actuation may help address diagnostic challenges. This opportunity is relevant for handling trace biomolecular solutions in picoliter–microliter volumes [450], where reagent conservation strongly influences analytical cost and throughput. A representative example is droplet digital PCR (ddPCR) [451], which partitions trace nucleic‐acid samples into large numbers of microdroplets for sensitive and quantitative detection. Such systems are highly sensitive to environmental and externally imposed conditions, including temperature, humidity, evaporation, electromagnetic exposure, and even acoustic perturbations. Many bioanalytical technologies therefore already incorporate strong field‐mediated control, such as capillary electrophoresis [452, 453], where robust operation often depends on careful regulation, calibration, or compensation of field‐dependent transport processes.

In this context, hybrid strategies offer a route toward simplified yet controllable biomolecular liquid handling. For trace‐volume platforms such as ddPCR [451], passive interfacial designs may help suppress evaporation, reduce nonspecific adsorption, and stabilize droplet confinement, thereby improving nucleic‐acid retention and assay reproducibility while lowering actuation demand. Thermo‐responsive materials such as PNIPAM, with a transition temperature ($∼32^{\circ }C$) near physiological conditions [454], may further provide biologically compatible adaptive control. Combined with programmable field‐mediated operations, such hybrid designs could enhance robustness in biomolecular diagnostics performed under fluctuating environmental and sample conditions. Integrating digital control logic with scalable interfacial engineering could therefore enable diagnostic platforms that adapt to varying sample properties and operational requirements, facilitating integrated, field‐deployable, and user‐friendly systems capable of executing complex assays with minimal manual intervention.

Section 6.3 examines the role of liquid manipulation in healthcare engineering. Classic microfluidic technologies have advanced biomedical applications [455, 456, 457, 458]; in contrast, this review concentrates on interfacial liquid control along with various dynamic interactions. Looking ahead, we highlight the critical need for hybrid liquid control strategies to address clinical challenges and advance personalized healthcare.

### 6.4 Other Interdisciplinary Applications

Interfacial liquid manipulation has expanded into diverse interdisciplinary applications. For example, asymmetric surfaces enabling multidirectional liquid control can conversely function as portable surface tension meters [119]; balanced liquid–solid interfacial energy, quantified by a dimensionless parameter ζ, has been used to develop liquid‐based encryption systems [47], where concealed information is revealed through specific liquid–surface interactions; surface‐energy‐patterned stamps allow “one stamp, diverse patterns” fabrication by exploiting the Cassie–Wenzel wetting transition of inks with tailored surface tensions [459]. Beyond these, biomimetic interfacial engineering has inspired functional designs such as spill‐resistant containers [460] and noise‐suppressing elastomers [461]. These examples further illustrate that the selection of passive, active, or hybrid strategies depends strongly on application demands. Passive approaches are attractive for systems prioritizing simplicity, portability, and autonomous operation, whereas active methods are favored when real‐time tunability or externally responsive control is required. Hybrid strategies provide an intermediate route by combining passive structural guidance with controllable actuation, making them particularly suitable for multifunctional and adaptive systems that seek both operational flexibility and reduced energy consumption. Together, these developments highlight how interfacial liquid control drives innovations across sensing, secure interfaces, manufacturing, and practical everyday technologies.

Section 6 examines the applications of liquid manipulation in sustainable energy, smart manufacturing, healthcare engineering, and interdisciplinary scenarios. The integration of passive interfacial engineering with active external control has become a prominent paradigm. However, the practical deployment of such hybrid systems must balance added complexity, cost, and energy requirements against the functional gains they provide. We envision that two deployment pathways are particularly feasible: (1) high‐value, small‐scale applications, where the complexity and cost from external control are justified by substantial gains, for example, targeted therapeutics and bespoke diagnostics; (2) large‐scale, energy‐autonomous applications, where active components are powered by readily available sources (e.g., solar, wind, or ambient vibration), enabling robust and continuous operation in actual applications. By integrating responsive materials, external actuation, and interfacial architectures, future liquid manipulation systems can evolve from static and single‐function designs toward adaptive and multifunctional platforms. Such advances will address existing technological challenges and open pathways to sustainable, efficient, and intelligent liquid management solutions across science, engineering, and society.

## 7 Conclusions and Perspectives

This review has surveyed liquid manipulation from foundational interfacial theories and design principles to its diverse applications. The field has progressed substantially, advancing from passive strategies that harness intrinsic interfacial energy to active methods driven by external fields. Within this landscape, we highlight emerging hybrid systems that synergize elements belonging to passive and active approaches, for example, responsive materials, micro/nano‐structured architectures, and chemically tunable interfacial wettability, to enhance adaptability and intelligence. In this final section, we analyze key challenges and opportunities spanning energy utilization, material innovation, design methodology, and system integration to provide a forward‐looking perspective for next‐generation liquid handling technologies.

### 7.1 Energy Focus: Interfacial Fundamentals and Beyond

At the heart of any liquid manipulation strategy lies the sourcing, application, and conversion of energy. Future trajectories will move beyond singular energy modalities toward sophisticated energy management paradigms. One direction involves multi‐field coupling, where future systems will exploit the synergy of multiple external fields with intrinsic interfacial energy, that is, hybrid liquid manipulation solutions. The challenge here is not merely simultaneous application but intelligent coupling, which can unlock powerful synergistic functions while adhering to the principles of simplicity and low‐cost implementation. As manipulation targets shrink to nanoliter volumes, precision energy control becomes paramount, demanding spatiotemporal precision of energy input [462, 463]. The goal is to achieve minimally invasive control, where external energy is applied in a just‐in‐time and just‐enough manner, preserving the integrity of sensitive biological or chemical samples within the liquid. Looking further ahead, high‐efficiency and sustainable energy conversion will be key to developing ambient energy harvesters for sustainable and autonomous liquid manipulation [464, 465, 466]. This requires efficient energy‐conversion materials and designs, such as photothermal surfaces that maximize light‐to‐heat conversion. Beyond harvesting, mastering energy recovery—for instance, from capillary release during droplet coalescence—remains crucial for creating fully energy‐self‐sufficient fluidic systems.

### 7.2 Material Focus: Responsive and Multifunctional Composites

Advanced materials constitute the enabling platform for liquid manipulation, with future development directed toward systems that are not merely responsive, but multi‐modal, composite, and dynamically reconfigurable. A key direction involves multiresponsive and cascade‐coupled materials: beyond single‐stimulus responsiveness, future materials should respond to multiple stimuli (such as temperature and light, or electric fields and pH), and more sophisticatedly, engineer internally coupled responses that cascade across different physical or chemical domains, enabling rich liquid manipulation behaviors with minimal external input. To realize such capabilities, the ideal hybrid manipulation interface should manifest as a robust, multifunctional composite that seamlessly integrates special wettability characteristics, whether superhydrophobic, superhydrophilic, or slippery, with strong responsiveness to external fields. The central challenge here lies in preserving functional integrity over time and ensuring that the material's responsiveness does not compromise its designed wettability, and vice versa, under repeated or prolonged actuation. Besides, the frontier of interfacial design resides in dynamically reconfigurable properties, surpassing today's predominantly static or binary‐switchable architectures. By leveraging advanced materials such as nanoscale textures, liquid crystal polymers, or shape‐memory alloys, future surfaces could continuously tune their wettability, adhesion, or topography in response to external stimuli [467]. In this context, a single platform could perform diverse liquid‐handling tasks on demand, simply by altering its own properties.

### 7.3 Design Focus: Passive Structures and Active Architectures

Surface structural design serves as the cornerstone of passive manipulation and the structural backbone for integrating active components. Future design evolution must shift from static mimicry toward dynamic and intelligent frameworks. This begins with next‐generation bioinspired architectures: rather than directly replicating natural structures such as lotus leaves or pitcher plants, future design must embrace deconstructive inspiration, extracting underlying principles (e.g., capillary action, hierarchical roughness, surface chemistry) and recombining them with engineering constraints and objectives to create principled architectures that are manufacturable, scalable, and tunable [468, 469]. As the complexity of micro/nano‐topography and chemical patterning escalates, particularly when combined with active actuation, the limitations of intuition and trial‐and‐error become apparent. At this juncture, artificial intelligence (AI), including machine learning and generative models, proves indispensable [470, 471]. By training on data that correlate structural parameters with wetting and transport behavior, AI enables the inverse design of surfaces for targeted liquid performance, potentially uncovering non‐intuitive solutions and optimizing competing objectives [472, 473]. Ultimately, because hybrid liquid manipulation hinges on the nexus between passive design and active control, the core challenge lies in structural‐dynamic optimization: co‐optimizing static structural parameters with dynamic field parameters to create architectures that establish a favorable passive energy landscape while simultaneously amplifying and guiding active stimuli. This demands coupled multiphysics models that treat the structure and stimulus as an integrated system.

### 7.4 System Focus: Toward Integrated Intelligent Liquid Manipulation

The ultimate measure of progress in liquid manipulation lies in the realization of robust, deployable systems that solve real‐world problems [474, 475], with the integration of passive and active strategies serving as a pivotal foundation for building such powerful and practical platforms. At the operational level, future systems must transition from demonstrating isolated single functions to executing complex, preprogrammed protocols comprising multiple sequential operations. This requires not only hardware advancements but also integrated sensor feedback for real‐time monitoring of droplet position, volume, and composition, coupled with closed‐loop control algorithms that ensure reliable execution. Beyond operational complexity, future platforms should transcend today's single‐function chips to post‐fabrication reconfigurability [476, 477]. By enabling platform functions to be altered through software (e.g., reprogrammable field patterns) or hardware (e.g., modular components), such designs will improve versatility, adaptability, and long‐term cost‐effectiveness. Ultimately, the trajectory of development must be application‐driven, targeting domains where current technologies are inadequate [478, 479, 480, 481], for example, the development of lab‐on‐a‐chip diagnostics that perform complex assays; advanced thermal management systems for high‐power electronics; water harvesting surfaces that optimize condensation using solar‐driven processes; and additive manufacturing techniques that precisely deposit functional micro‐droplets. In all areas, successful translation from laboratory demonstrations to practical deployment requires that designs prioritize cost‐effectiveness, durability, energy efficiency, sustainability, and ease of use from the earliest stages of development.

In conclusion, we conceive that the future of liquid manipulation lies in its hybrid evolution, where the elegance of passive control and the precision of active methods converge into a unified paradigm. This approach unlocks a new generation of adaptive, intelligent, and efficient systems. Achieving this vision requires sustained collaboration across disciplines and a consistent focus on translating fundamental insights into practical and impactful technologies.

## Conflicts of Interest

The authors declare no conflicts of interest.

## Data Availability

Data sharing not applicable to this article as no datasets were generated or analyzed during the current study.
